# Striatal Molecular Signature of Subchronic Subthalamic Nucleus High Frequency Stimulation in Parkinsonian Rat

**DOI:** 10.1371/journal.pone.0060447

**Published:** 2013-04-04

**Authors:** Sylviane Lortet, Emilie Lacombe, Nicolas Boulanger, Pascal Rihet, Catherine Nguyen, Lydia Kerkerian-Le Goff, Pascal Salin

**Affiliations:** 1 Aix-Marseille Université, CNRS, IBDM UMR 7288, Marseille, France; 2 Aix-Marseille Université, INSERM, TAGC U1090, Marseille, France; Karolinska Institute, Sweden

## Abstract

This study addresses the molecular mechanisms underlying the action of subthalamic nucleus high frequency stimulation (STN-HFS) in the treatment of Parkinson's disease and its interaction with levodopa (L-DOPA), focusing on the striatum. Striatal gene expression profile was assessed in rats with nigral dopamine neuron lesion, either treated or not, using agilent microarrays and qPCR verification. The treatments consisted in anti-akinetic STN-HFS (5 days), chronic L-DOPA treatment inducing dyskinesia (LIDs) or the combination of the two treatments that exacerbated LIDs. STN-HFS modulated 71 striatal genes. The main biological processes associated with the differentially expressed gene products include regulation of growth, of apoptosis and of synaptic transmission, and extracellular region is a major cellular component implicated. In particular, several of these genes have been shown to support survival or differentiation of striatal or of dopaminergic neurons. These results indicate that STN HFS may induce widespread anatomo-functional rearrangements in the striatum and create a molecular environment favorable for neuroprotection and neuroplasticity. STN-HFS and L-DOPA treatment share very few common gene regulation features indicating that the molecular substrates underlying their striatal action are mostly different; among the common effects is the down-regulation of *Adrb1*, which encodes the adrenergic beta-1- receptor, supporting a major role of this receptor in Parkinson's disease. In addition to genes already reported to be associated with LIDs (preprodynorphin, thyrotropin-releasing hormone, metabotropic glutamate receptor 4, cannabinoid receptor 1), the comparison between DOPA and DOPA/HFS identifies immunity-related genes as potential players in L-DOPA side effects.

## Introduction

The degeneration of nigrostriatal dopamine neurons is a main pathological feature of Parkinson's disease (PD) [1]. This leads to alterations in the basal ganglia and hence to the motor disabilities characterizing PD, such as akinesia [2]. The dopamine replacement therapy using levodopa (L-DOPA) is the gold standard pharmacological treatment of PD. After few years, the great efficacy of this treatment is most often compromised by the development of disabling motor complications that gradually worsen, including motor fluctuations and L-DOPA-induced dyskinesias (LIDs). Progress in the knowledge of the mechanisms underlying LIDs, in particular of the cellular and molecular changes occurring in the striatum, the main input station of the basal ganglia, have opened new perspectives for their prevention or alleviation [3]–[6].

The functional neurosurgical treatment by subthalamic nucleus high frequency stimulation (STN-HFS) has demonstrated its efficacy to alleviate main PD motor symptoms, allowing in most cases a radical decrease of dopaminergic medication [7]. STN-HFS then attenuates LIDs indirectly by reducing L-DOPA requirement. There is evidence that this surgical treatment may not have a direct antidyskinetic action. For instance, when combined to dyskinesiogenic L-DOPA treatment, STN-HFS does not improve but can even exacerbate LIDs in PD patients and animal models [8]–[10]. As possible substrates, STN-HFS exacerbates L-DOPA-induced changes in several striatal markers associated to LIDs [10] and prolongs the increase in extracellular dopamine levels elicited by acute L-DOPA treatment [11]. There is accumulating evidence that the action mechanisms of STN-HFS are complex and that this surgical treatment might induce duration-dependent adaptive changes, within and outside the basal ganglia, and interfere with the disease progression [12]. In particular, we previously provided evidence that prolonged STN-HFS in PD model interferes with adult neurogenesis, promoting survival of newly formed cells in the regions of constitutive neurogenesis and also in the striatum [13]. Elucidating the mechanisms of STN-HFS and its interaction with L-DOPA is crucial, especially since up to recent trials, all PD patients selected for HFS had a long history of L-DOPA treatment. Although the striatum is not a primary target of STN, STN-HFS has been reported to impact glutamate, GABA and dopamine striatal neurotransmission, suggesting possible widespread action at striatal level [12]. However, little is known about the striatal molecular pathways implicated by STN-HFS.

A number of studies have examined gene regulation in post-mortem tissues of PD patients and in animal models, mostly in the substantia nigra but also in the striatum. The effects of L-DOPA treatment on striatal gene expression have been already addressed [14]–[18] whereas the effects of STN-HFS have not been investigated. In this study, gene array technology was used to perform a large scale screening of the genes with altered striatal expression in a rat PD model after antiakinetic subchronic STN-HFS and L-DOPA treatment inducing dyskinesias, either separately or in combination, in comparison with lesioned animals without any treatment. The objectives were 1) to identify the molecular signature of STN-HFS action at striatal level, associated with its efficient antiparkinsonian action, and 2) to investigate the molecular substrates of the interaction between the two treatments in order to evidence possible genes involved in LIDs as STN-HFS exacerbates LIDs. The pattern of genes modulated under STN-HFS suggests that the neurosurgical treatment generates a molecular environment that favors growth, neuroplasticity and neuroprotection. Whereas wide changes occur after STN-HFS or L-DOPA, few common regulations are found. Finally, comparison of the genes modulated in the 3 experimental conditions suggests an association between immunity-related genes and LIDs.

## Materials and Methods

### Experimental groups

All animal experimental procedures were carried out in strict accordance with local rules concerning the use of laboratory animals (authorization #B 13-267) and with the recommendations of the EEC (86/609/EEC) for care and use of laboratory animals, and conformed to the ethical guidelines of the French Ministry of Agriculture and Forests (Animal Health and Protection Veterinary Service). All animals used in this study were maintained on a 12∶12-h light/dark cycle (lights on: 7:00 A.M. to 7:00 P.M.), with food and tap water available ad libitum and every precaution was taken to minimize the stress and the number of animals used in each series of experiments. During surgery, wounds and pressure points were repeatedly (every 2 h) infiltrated with lignocaine (2%). Postoperative analgesia was performed by adding Acetaminophen (400 mg/kg) in the drinking water during 7 days.

Microarray experiments were carried out on striatal tissue from four groups of animals bearing 6-hydroxydopamine (6-OHDA)-induced lesion of the nigrostriatal DA pathway: lesion alone without any subsequent treatment (6-OHDA), dyskinesiogenic L-DOPA treatment for 19 days (DOPA), STN-HFS for 5 days (HFS) and combination of dyskinesiogenic L-DOPA and STN-HFS (DOPA/HFS); in this last group HFS was applied during the 5 last days of the 19-days L-DOPA treatment. Quantitative RT-PCR was performed on striatal tissue from 2 additional groups of lesioned animals either stimulated or not, 6-OHDA group and HFS group, and a group of unlesioned rats with an electrode implanted (control). Each experimental group for the microarray study contained 3 animals, and for RT-qPCR 4 to 5 animals.

### Surgery and treatment

Surgery was performed under Equithesin anaesthesia (4 ml/kg). Animals received a unilateral injection of 9 µg of 6-OHDA (Sigma-Aldrich, St Quentin–Fallavier, France) dissolved in 4.5 µL of 0.9% sterile NaCl containing 0.1% ascorbic acid, at a rate of 1 µL/min, in the left SNc. The stereotaxic coordinates of the injection site were: anteroposterior +2.2 mm, lateral 2.0 mm and dorsoventral +3.3 mm from the interaural, with the incisor bar at +5.0 mm above the interaural plane, according to the rat stereotaxic atlas by [19]. These animals were killed by decapitation 33 days after 6-OHDA lesion. Fourteen days after 6-OHDA lesion, two groups of rats (DOPA and DOPA/HFS) received chronic L-DOPA treatment for 19 days. This treatment consisted of two injections per day, at a 12-h interval, of 25 mg/kg L-DOPA and 12.5 mg/kg benserazide (Sigma–Aldrich) dissolved in 0.9% NaCl. All L-DOPA-treated animals, with or without HFS, received the last injection 12 h before killing by decapitation. Fourteen days after the 6-OHDA lesion, the groups of rats to be treated by STN-HFS (HFS and DOPA/HFS) were anaesthetized by Equithesin i.p. injection and were unilaterally implanted with one platinum-iridium electrode for deep brain stimulation in the STN ipsilateral to the lesion side as described previously [10]. The stereotaxic coordinates were taken averaging the interaural and bregma coordinates [20]; from bregma, they were: anteroposterior −3.8 mm (equidistant from the two wires), lateral 2.4 mm, dorsoventral −8.1 mm from the surface of the meninges. STN-HFS was applied continuously for 5 days in freely moving rats, starting 28 days after 6-OHDA lesion, under conditions proven efficient for alleviating DA denervation-mediated akinesia [9], [10]. Stimuli were delivered by a pulse generator and a stimulus isolation unit (P2MP, Marseille, France), which gave rectangular current pulses. The stimulation parameters were: frequency: 130 Hz; pulse width 80 µs; intensity 60 µA (which was about twice below the threshold intensity provoking dyskinetic movements of the contralateral forepaw [10]). HFS and DOPA/HFS animals were killed by decapitation immediately after turning off the stimulation.

### Behavioral assessments

Animals of the control, 6-OHDA and HFS groups were scored for akinesia of the contralateral forelimb by using the cylinder test. In brief, animals were placed in a Plexiglas cylinder and, immediately after, videotaped for 30 min to examine the symmetry/asymmetry of their forepaw use during their explorative behavior in this new environment. The numbers of contacts made on the cylinder wall during this period with the ipsilateral paw, the contralateral paw, and both paws (double contacts) were determined; a double contact was scored as an ipsilateral contact plus a contralateral contact. Akinesia was represented as forelimb asymmetry, i.e. % contralateral minus % ipsilateral forepaw contacts.

For quantification of LIDs, animals of the DOPA and DOPA/HFS groups were videotaped for 2 h after the injection of L-DOPA at day 14 and day 19 of the chronic treatment, which corresponded respectively to the time point just before starting STN-HFS and the last day of HFS treatment. Individual animals were scored for 1 min every 10 min, from 10 to 120 min after the injection of L-DOPA as reported previously [21]. Axial, orolingual, and forelimb dyskinesias were scored using a scale from 0 to 4 as defined previously [22].

### Tissue preparation

Animals were killed by decapitation, the brain quickly removed, immediately frozen on dry ice powder and stored at −80°C until use. Brains were sectioned at −20°C using a cryostat. Part of the striatum was cut in thin sections (12 µm) for histological analysis. On the other part, the dorsal striatum was punched from thick sections and samples pooled to obtain around 10 mg tissue for RNA extraction. Thin 12 µm sections were also performed at STN level to control the electrode placement.

### Control of the DA lesion extent and electrode implantation site

The loss of DA terminals in the striatum was assessed as an index of the extent of the DA denervation by analysis of ^3^H-mazindol binding to DA uptake sites on film autoradiograms, as described previously [23]. The location of the stimulating electrode in the STN was examined on fresh cryostat sections. Animals showing a reduction of less than −85% in 3H-mazindol binding or a misplaced electrode were not included in the experimental groups presented above.

### Transcriptomic analysis and reverse transcriptase quantitative PCR (RT-qPCR)

Total RNA was extracted using the RNeasy Mini kit (Quiagen, Courtaboeuf, France) according to the manufacturer's instructions. RNA concentration was determined with a nanodrop ND-1000 spectrophotometer (nanodrop technologies, Wilmington, DE, USA) and the quality was evaluated with an Agilent bioanalyzer (Agilent technologies, Santa Cruz, CA, USA). The RNA samples selected for microarray and for real-time PCR experiments had an >8.0 RNA integrity number on a scale of 10. RNA was stored at −80°C until use.

For microarray experiments, amplification and fluorescent labeling of RNA was performed with the kit LIRLAK one color (Low Input RNA Linear Amplification Kit, Agilent) with starting amounts of 200 to 400 ng of total RNA. cRNAs were hybridized on Rat AGILENT 4×44 K microarrays and, after adequate washing, scanned with Agilent Microarray Scanner. All steps were performed according to the manufacturer (Agilent Technologies, Santa Cruz, CA, USA). All individual samples were hybridized onto separate microarrays. Thus, 12 Agilent oligonucleotide microarrays were used to compare the gene expression profiles of 6-OHDA, HFS, DOPA and DOPA/HFS groups. Microarray data were extracted and quantified with Feature extraction software (Agilent). The AgiND R library was further used for data diagnosis and normalization (http://tagc.univ-mrs.fr/tagc/index.php/software/16). Diagnosis was based on boxplot, color-coded images and MA plot, while normalization was performed based on the quantile method. Statistical analysis was performed using the TIGR MeV (MultiExperiment Viewer) v4.1 software (http://www.tm4.org/mev.html) and the GeneANOVA programs. First, a global ANOVA model gives an estimation of the contribution of HFS and DOPA in the total variation of the whole data set; gene was considered a factor in this model [24]. Genes showing significant differential expression between the 4 experimental groups were determined by a multi-class analysis using Significance Analysis of Microarrays (SAM) of TMev with 100 permutations. Hierarchical cluster analysis was applied to these genes in order to identify samples with similar profiles. Genes with >1.2 or <0.8 fold change in the HFS, DOPA and DOPA/HFS groups compared to 6-OHDA were further analyzed using two statistical tests. A two-class unpaired SAM of TMev with 20 permutations was first performed, with adaptive false discovery rate (FDR) procedure [25]. FDR was <10% for HFS and DOPA (6 and 1%, respectively) and was 13% for DOPA/HFS due to individual variability in this group. From these genes, those which did not reach significance with a second statistical test, the non-parametric Wilcoxon test (with p<0.05) were then subtracted. Annotation of the significantly altered genes was performed using DAVID database (http://david.abcc.ncifcrf.gov/) [26]. This program was used to assess whether gene ontology (GO) terms and the Kyoto Encyclopaedia of Genes and Genomes (KEGG) pathways were overrepresented among the differentially expressed genes and within specific gene clusters. A score based on Fisher's exact test reflected the probability that the prevalence of a particular term within a cluster was due to chance alone, given the prevalence of that term in the population of all genes under study.

For RT-qPCR validation of microarray data, six of the genes showing differential expression in the HFS vs 6-OHDA group by microarray analysis and an endogenous reference gene, hypoxanthine phosphoribosyltransferase (HPRT) were evaluated in control, 6-OHDA and HFS groups. Gene specific primers were designed using Universal ProbeLibrary (ProbeFinder version 2.45 for rat, Roche Diagnostics) and chosen intron-spanning when possible (Table S1). Total RNA (0.5 µg) was reverse transcribed for 60 min at 42°C using ImProm-IITM reverse transcriptase (Promega, Charbonnières-les-Bains, France) with random hexamers. Real-time qPCRs were carried out in a total volume of 20 µl using 1/100 of the cDNA produced by reverse transcription, SsoFast EvaGreen Supermix (BioRad) and primer pair of the gene of interest (eurofins operon) using Bio-Rad CFX96 cycler with the following cycling parameters: 1 cycle at 95°C for 30 s (enzyme activation); 40 cycles at 95°C for 10 s followed by 60°C for 30 s (denaturation and annealing). Samples were run in duplicate for each gene analyzed. Primer pairs were tested for specificity with the melting curve analysis (0.5°C increment from 55°C to 95°C). PCR efficiency laid around 110% for all the primers used. Ct values for the reference and for the target genes were in similar ranges and Ct for the reference was not changed among experiments; furthermore HPRT expression analyzed by microarray also revealed no difference between all the samples, confirming the validity of HPRT as a reference gene. Several no-template controls, which produced no signal, were also included. Relative target gene concentration was calculated using the 2^−ΔΔCt^ method which uses normalization to endogenous reference gene and normalization to calibrator sample (here, mean of the 3 values of the 6-OHDA group). Data were analyzed by ANOVA with a Fisher test. The differences were defined as significant when p<0.05.

## Results

### Control of the 6-OHDA-induced denervation extent in the striatum and of the electrode location in STN

**Figure 1:**
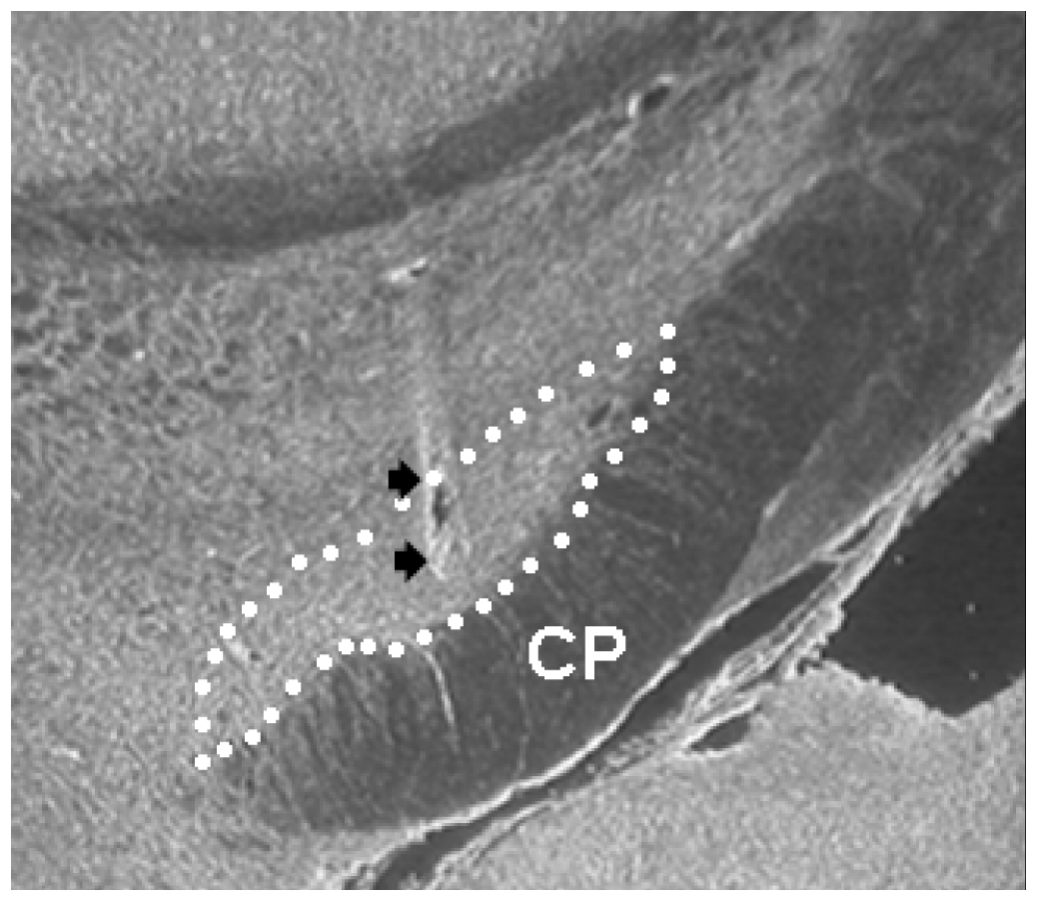
Photomicrograph illustrating the electrode placement in the STN. Subthalamic nucleus was outlined by the white dotted line. The black arrows show the location of the bared electrode tips delivering HFS in the STN. CP, cerebral peduncle.

The selected 6-OHDA animals included in the four experimental groups showed an extensive striatal dopamine denervation, as assessed by the marked loss of ^3^H-mazindol binding in the ipsilateral striatum vs controls (6-OHDA: −91.29±2.76%; HFS: −88.32±3.03%; DOPA: −95.03 ±0.92%; DOPA-HFS: −90.66±2.19%). Figure 1 illustrates the correct location of the stimulating electrode in the STN in the selected animals.

### Behavioral observations

**Figure 2:**
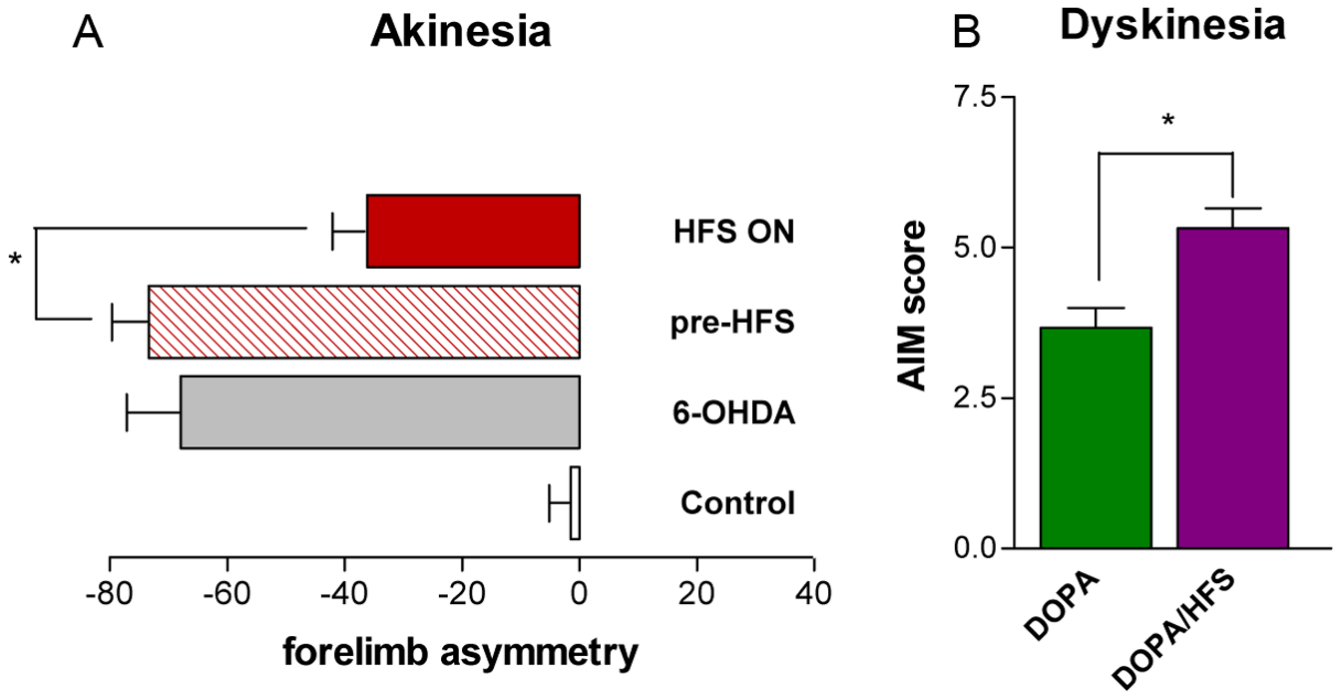
STN HFS alleviates akinesia and exacerbates L-DOPA-induced dyskinesia. (A) Akinesia was assessed by forelimb asymmetry in the cylinder test in the control and 6-OHDA groups and in the HFS group before the stimulation (pre-HFS) and after 5 days of stimulation (HFS-ON). Forelimb asymmetry was calculated as the % contralateral minus % ipsilateral forepaw contacts (*p<0.05 HFS ON vs. pre-HFS). (B) Analysis of AIMs was performed in DOPA and DOPA/HFS groups. Dyskinesia score corresponds to the sum of the orolingual and forelimb AIM scores (maximum 8). The data are the means ± SEM, *p<0.05 DOPA/HFS vs DOPA.

In the cylinder test, control animals equivalently use their right and left forepaws (Figure 2A). Animals of the 6-OHDA group and of the HFS group tested before starting the stimulation (pre-HFS) showed a marked asymmetry, the number of contralateral contacts being markedly reduced due to akinesia. This deficit is partially but significantly reversed after 5 days of HFS in the HFS group (HFS ON).

L-DOPA-treated animals in the DOPA and the DOPA/HFS groups presented almost no axial dyskinesias, so that scores were established based on orofacial and forelimb dyskinesias. The dyskinesia score (Figure 2B) was relatively low in the DOPA group (3.67±0.58) and was significantly higher in the DOPA/HFS group (5.33±0.58) in agreement with previous data showing that STN-HFS exacerbates LIDs [10], [27].

### Global gene analysis of the four groups: 6-OHDA, HFS, DOPA and DOPA/HFS

We performed a gene profiling study using 44000 oligonucleotide microarrays to investigate the striatal effect of STN-HFS, of DOPA treatment and of the combined STN-HFS/DOPA treatment. The data obtained have been deposited in NCBI's Gene Expression Omnibus and are accessible through GEO Series accession number GSE43375 (http://www.ncbi.nlm.nih.gov/geo/query/acc.cgi?acc=GSE43375).

**Figure 3:**
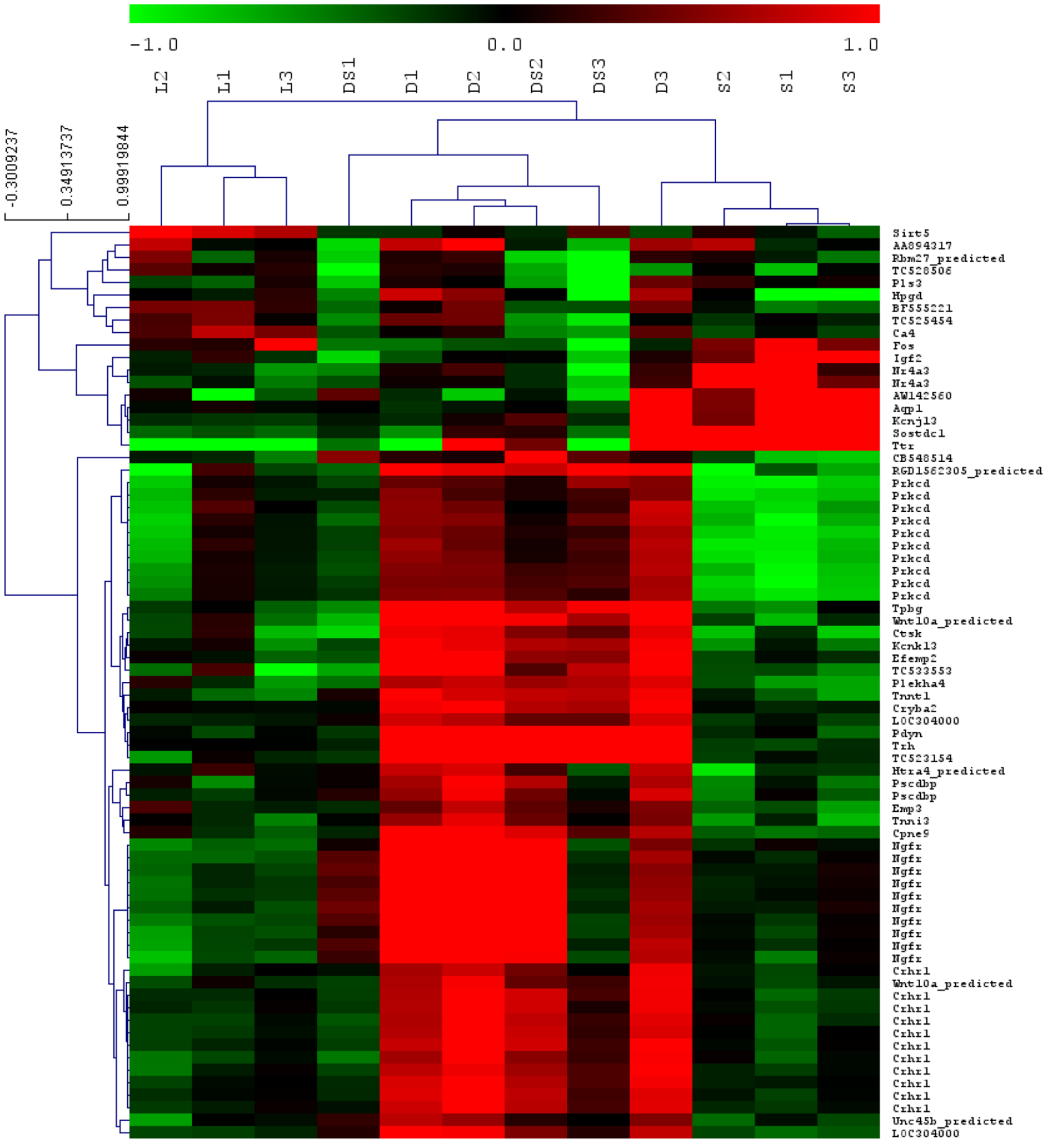
Hierarchical clustering. The 12 experimental striatal samples were: L1, L2 and L3 (6-OHDA); S1, S2 and S3 (HFS); D1, D2 and D3 (DOPA) and DS1, DS2 and DS3 (DOPA/HFS). The hierarchical clustering was constructed with the genes showing significant changes after a 4 group Sam analysis on the genes with expression value above detection limit in at least 40% of the samples. Upregulated and down-regulated genes appear in red and in green, respectively, with the relative log2 (ratio) reflected by the color intensity. Three sample clusters were found: 6-OHDA, DOPA with DOPA/HFS and HFS.

The global gene ANOVA analysis demonstrated a very significant effect of the different treatments with p value less than 10^−5^ (Table S2). Hierarchical cluster analysis (Figure 3) was used to test if changes in gene expression levels segregate the experimental groups. It showed two clusters of samples with similar gene profiles: one regrouped the samples from the 6-OHDA animals without treatment (L) and the other gathered together the samples from all the treated animals. The samples from treated animals further clustered in two sub-groups, the first one including the DOPA and DOPA/HFS samples (D and DS, respectively, except one dopa-treated animal) and the second one the three HFS samples (S). These results demonstrate that HFS and L-DOPA treatments strongly affect gene expression in the striatum and that the gene expression profile in the combined treatment condition was close to that of DOPA treatment alone and not to that of HFS alone.

### Genes differentially expressed in HFS, DOPA and DOPA/HFS versus 6-OHDA

**Figure 4:**
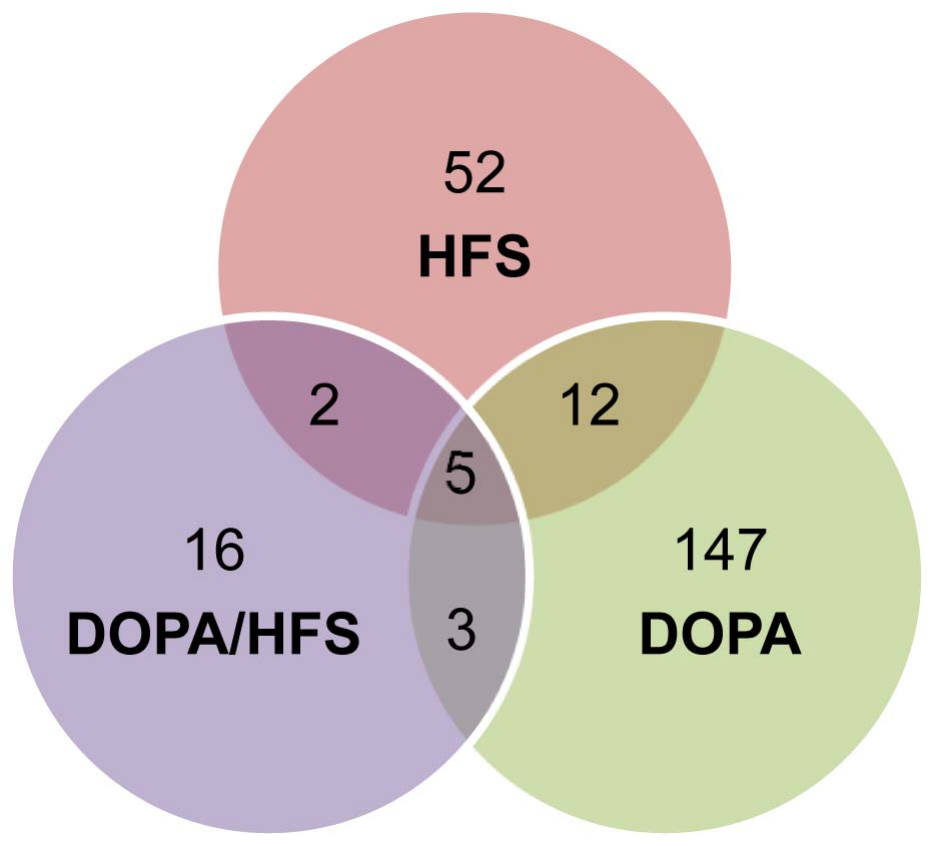
Venn diagram showing the numbers of genes specifically or commonly altered by the treatments.

The numbers of genes differentially expressed in each of the three treatment groups as compared to 6-OHDA are presented in Figure 4. Of the 71 genes showing differential expression in the HFS group (listed in Table S3), twice as many were down-regulated than up-regulated. More genes, 167, were affected in the DOPA group (Table S4), with equivalent numbers being down-regulated and up-regulated. Because of individual variability, only 26 genes were found in the DOPA/HFS group (Table S5), most being down-regulated. To note however that several of the genes induced by L-DOPA and reported to be associated with LIDs, such as *Pdyn* (preprodynorphin) and *Trh* (thyrotropin-releasing hormone) show a clear tendency towards upregulation in the DOPA/HFS group (respectively 1.6±0.5 and 2.5±1.4 fold change vs 6-OHDA).

Only five genes were commonly regulated by all the treatments, 3 being down-regulated: *Adrb1* (β1-adrenergic receptor), *Btg2* (B-cell translocation gene 2), and *Sirt5* (sirtuin 5), and 2 up-regulated: *Galntl5* (polypeptide N-acetylgalactosaminyltransferase-like 5) and *Ngfr* (Nerve growth factor receptor). Two more genes were found in common for HFS and DOPA/HFS: *Carhsp1* (calcium-regulated heat stable protein 1) and *Ebf1* (early B-cell factor 1), 3 for DOPA and DOPA/HFS: *C1s* (complement C1s subcomponent), *Rt1-Da* (RT1 class II, locus Da) *and Irf7* (interferon regulatory factor 7) and 12 for HFS and DOPA: *bbc3* (bcl-2 binding component 3, also called PUMA), *emp3* (epithelial membrane protein 3), *geft/Arhgef25* (rho guanine nucleotide exchange factor 25), *itpka* (Inositol-1,4,5-trisphosphate 3-kinase A), *loc683470* (similar to growth arrest specific 1), *loc684626* (similar to K11B4.2), *loc688966/Mef2bnb* (MEF2B neighbor), *nr4a3* (nuclear receptor subfamily 4 group A member 3), *prkcb* (protein kinase C beta type), *prkcd* (protein kinase C delta type), *slc18a2* (vesicular monoamine transporter 2), *trpc4* (short transient receptor potential channel 4). To note that *emp3* and *PrKcd* were inversely regulated by HFS and by DOPA.

**Figure 5:**
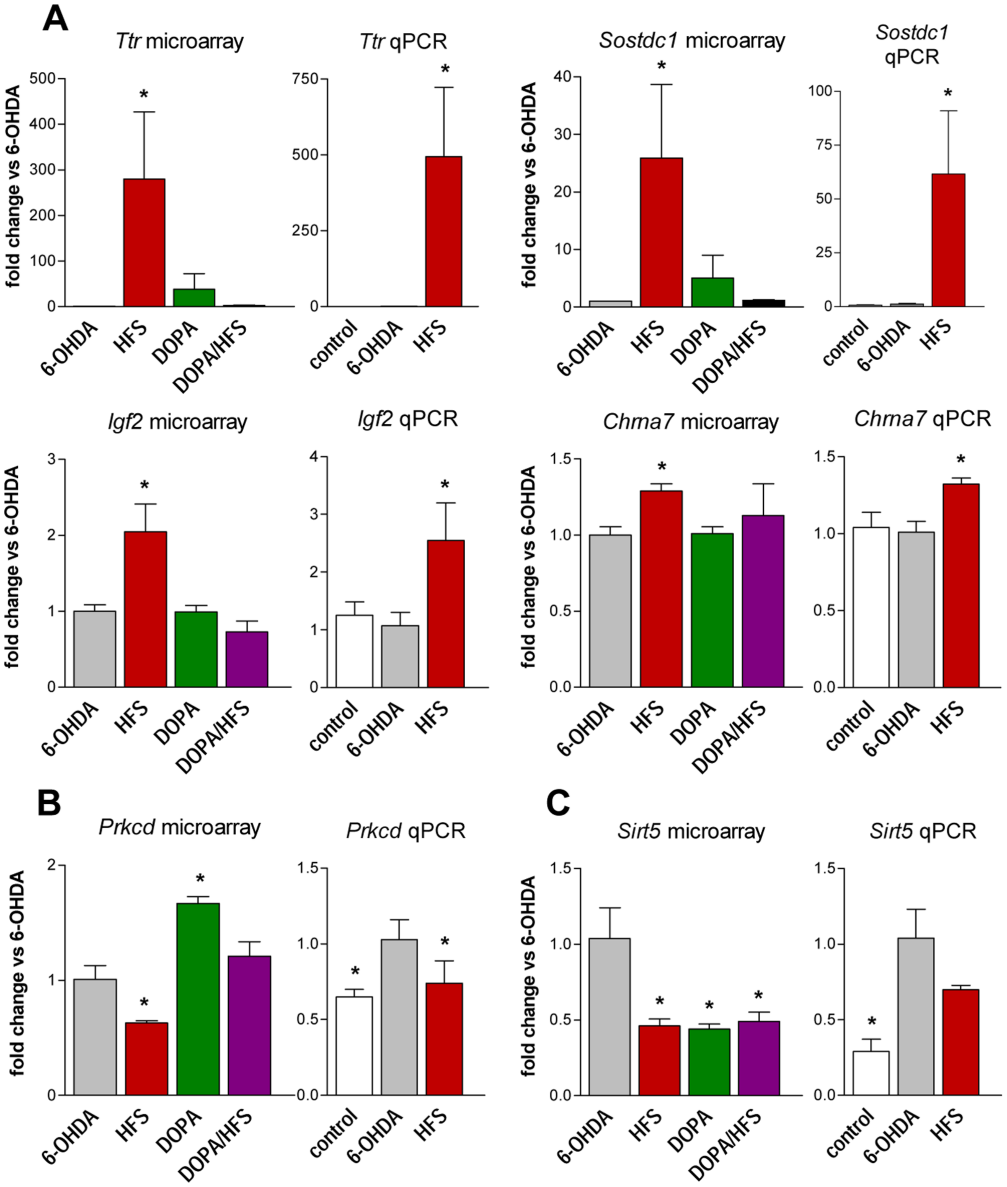
Comparison of the data obtained by microarray and RT-qPCR for 6 genes of interest. (A) Genes specifically up-regulated by HFS, (B) opposite regulation of *Pkrcd* by HFS and DOPA and (C) common down-regulation of *Sirt5* by HFS and DOPA. Microarray experiments were performed on total striatal RNA samples from 6-OHDA, HFS, DOPA and DOPA/HFS groups. For qPCR validation of gene expression, total striatal RNA samples from 3 groups were analyzed: control, 6-OHDA and HFS. For both microarray and qPCR, results were calculated for each sample relative to the expression of the endogenous reference gene: HPRT, and fold change vs the 6-OHDA group was determined using the 2^−ΔΔCt^ method. Values are presented as means ± SEM. *p<0.05 vs 6-OHDA values.

From the genes significantly altered in HFS group vs 6-OHDA (Table S3), 5 displayed a more than 2-fold increase: *Ttr* (transthyretin), *Sostdc1* (sclerostin domain containing 1), *Aqp1* (aquaporin 1), *Nr4a3* and *Igf2* (insulin growth factor 2); *Ttr* and *Sostdc1* showing the highest fold change: 279 and 26, respectively. The up-regulation of *Ttr, Sostdc1* and *Igf2* was verified by RT-qPCR (Figure 5A). RT-qPCR also confirmed the more modest (1.4-fold) up-regulation of *Chrna7* (nicotinic receptor alpha 7 subunit) found in microarray analysis. The PCR analysis showed that these 4 genes were not differentially expressed in 6-OHDA and control conditions, and were thus up-regulated by HFS vs control. In the microarray, these 4 genes were not modified in DOPA and DOPA/HFS conditions, suggesting that part of the HFS effects was lost when combined to L-DOPA treatment. Genes that were down-regulated vs 6-OHDA in HFS condition included *Prkcd*, which, in agreement with previous data [14] was up-regulated in the DOPA group (Figure 5B). *Prkcd* expression was unaffected in the DOPA/HFS, suggesting an addition of the opposite effects of the two treatments. We validated by RT-qPCR the down-regulation of *Prkcd* by HFS vs 6-OHDA (Figure 5B); since the PCR experiment showed that the *Prkcd* gene expression was lower in control vs 6-OHDA, we concluded that HFS, in fact, normalized the expression of this gene. In microarrays, one of the genes showing the more marked down-regulation by HFS was *Sirt5*, a gene also down-regulated in the DOPA and DOPA/HFS groups (Figure 5C). The decrease of *Sirt5* by HFS did not reach significance (p = 0.10) in the RT-qPCR analysis; this analysis further indicated that this gene was overexpressed in 6-OHDA vs control, suggesting tendency towards normalization by the treatments. Overall, we found very similar fold change values by microarray and RT-qPCR analysis, although these measurements were made in different experimental series of animals.

### Biological analysis of genes altered by HFS, DOPA and DOPA/HFS

In order to understand the biological meaning of the gene lists found in the previous analysis, we used DAVID bioinformatics resources that allow the identification of the most relevant (overrepresented) biological terms/functions associated with a gene list and the clustering into functionally related gene groups on the basis of their associated biological processes (BP), cellular components (CC) and molecular functions (MF). For the genes differentially expressed after HFS, 127 functional groups were identified (Table S6). Table 1 presents 8 functional groups selected from this list, as being highly significant, representative of several categories, or presumably related to PD after a detailed bibliographic search. The BP GO terms were “response to endogenous stimulus” (12 genes), “ion transport” (12 genes), “regulation of growth” (9 genes), “positive regulation of apoptosis” (10 genes), “regulation of synaptic transmission” (6 genes), “regulation of BMP signaling pathway” (3 genes), and “regulation of MAPKKK cascade” (4 genes); the CC GO term was “extracellular region” (10 genes). PD-related genes included in these functional groups were *Ngfr*, *Igf2*, *Prkcd*, *Chrna7* and *Mapk8* (mitogen-activated protein kinase 8). *Ttr* and *Sostdc1*, the two genes that displayed the higher level of induction after STN-HFS, shared the same GO term “extracellular region”, in compliance with their role as extracellular regulators of multiple signaling pathways.

**Table 1:** Enriched functional categories (GO terms) for genes differentially expressed after HFS vs 6-OHDA, with the constituting genes and their fold change (FC).

*Gene symbol*	Gene name	FC
**GOTERM_BP, Response to endogenous stimulus, p value 3.10^−5^**		
*Aqp1*	aquaporin 1	3.3
*Nr4a3*	nuclear receptor subfamily 4, group A, member 3	2.7
*Igf2*	insulin-like growth factor 2	2.0
*Rpe65*	retinal pigment epithelium 65	1.4
*Mapk8*	mitogen-activated protein kinase 8	1.2
*Enpp1*	ectonucleotide pyrophosphatase/phosphodiesterase 1	0.8
*Slc18A2*	solute carrier family 18 (vesicular monoamine), member 2	0.8
*Cacna1b*	calcium channel, voltage-dependent, N type, alpha 1B subunit	0.8
*Tnfrsf11b*	tumor necrosis factor receptor superfamily, member 11b	0.7
*Gnb1*	guanine nucleotide binding protein (G protein), beta polypeptide 1; guanine nucleotide binding protein (G protein), beta polypeptide 4	0.7
*Btg2*	B-cell translocation gene 2, anti-proliferative	0.6
*Prkcd*	protein kinase C, delta	0.6
**GOTERM_BP, Ion transport, p value 1.2.10^−4^**		
*Aqp1*	aquaporin 1	3.3
*Slc4A2*	solute carrier family 4 (anion exchanger), member 2	1.5
*Chrna7*	cholinergic receptor, nicotinic, alpha 7	1.3
*Slc12a4*	solute carrier family 12 (potassium/chloride transporters), member 4	1.2
*Tcirg1*	T-cell, immune regulator 1, ATPase, H+ transporting, lysosomal V0 subunit A3	0.8
*Enpp1*	ectonucleotide pyrophosphatase/phosphodiesterase 1	0.8
*Trpc4*	transient receptor potential cation channel, subfamily C, member 4	0.8
*Prkcb*	protein kinase C, beta	0.8
*Slc39A8*	solute carrier family 39 (metal ion transporter), member 8	0.8
*Cacna1b*	calcium channel, voltage-dependent, N type, alpha 1B subunit	0.8
*Cacnb2*	calcium channel, voltage-dependent, beta 2 subunit	0.7
*Prkcd*	protein kinase C, delta	0.6
**GOTERM_BP, Regulation of growth, p value 3.10^−4^**		
*Htra1*	HtrA serine peptidase 1	1.4
*Agt*	angiotensinogen (serpin peptidase inhibitor, clade A, member 8)	1.3
*Enpp1*	ectonucleotide pyrophosphatase/phosphodiesterase 1	0.8
*Hsd3b7*	hydroxy-delta-5-steroid dehydrogenase, 3 beta- and steroid delta-isomerase 7	0.8
*Prkcb*	protein kinase C, beta	0.8
*Adrb1*	adrenergic, beta-1-, receptor	0.7
*Gpc3*	glypican 3	0.7
*Bbc3*	Bcl-2 binding component 3	0.5
**GOTERM_BP, Positive regulation of apoptosis, p value 5.5.10^−4^**		
*Pdia3*	protein disulfide isomerase family A, member 3	1.5
*Agt*	angiotensinogen (serpin peptidase inhibitor, clade A, member 8)	1.3
*Ngfr*	nerve growth factor receptor (TNFR superfamily, member 16)	1.3
*Mapk8*	mitogen-activated protein kinase 8	1.2
*Tradd*	TNFRSF1A-associated via death domain	0.8
*Aph1a*	anterior pharynx defective 1 homolog A (C. elegans); similar to Gamma-secretase subunit APH-1A (APH-1a) (Aph-1alpha) (Presenilin-stabilization factor)	0.7
*Prkcd*	protein kinase C, delta	0.6
*Bbc3*	Bcl-2 binding component 3	0.5
**GOTERM_BP, Regulation of synaptic transmission, p value 7.5.10^−4^**		
*Chrna7*	cholinergic receptor, nicotinic, alpha 7	1.3
*Agt*	angiotensinogen (serpin peptidase inhibitor, clade A, member 8)	1.3
*Cacna1b*	calcium channel, voltage-dependent, N type, alpha 1B subunit	0.8
*Adrb1*	adrenergic, beta-1-, receptor	0.7
*Edn1*	endothelin 1	0.7
*Bhlhe40*	basic helix-loop-helix family, member e40	0.5
**GOTERM_BP, Regulation of BMP signaling pathway, p value 4.9.10^−3^**		
*Sostdc1*	sclerostin domain containing 1	25.9
*Htra1*	HtrA serine peptidase 1	1.4
*Gpc3*	glypican 3	0.7
**GOTERM_CC, Extracellular region, p value 5.4.10^−3^**		
*Ttr*	transthyretin	278.7
*Sostdc1*	sclerostin domain containing 1	25.9
*Igf2*	insulin-like growth factor 2	2.0
*Agt*	angiotensinogen (serpin peptidase inhibitor, clade A, member 8)	1.3
*Prelp*	proline arginine-rich end leucine-rich repeat protein	1.2
*Enpp1*	ectonucleotide pyrophosphatase/phosphodiesterase 1	0.8
*Tnfrsf11b*	tumor necrosis factor receptor superfamily, member 11b	0.7
*Vtn*	vitronectin	0.7
*Edn1*	endothelin 1	0.7
*Gpc3*	glypican 3	0.7
**GOTERM_BP, Regulation of MAPKKK cascade, p value 1.5.10^−2^**		
*Igf2*	insulin-like growth factor 2	2.0
*Agt*	angiotensinogen (serpin peptidase inhibitor, clade A, member 8)	1.3
*Edn1*	endothelin 1	0.7
*Prkcd*	protein kinase C, delta	0.6

Abbreviations: GO: gene ontology; BP: biological process; CC: cell. component.

The most enriched GO terms found after DOPA and DOPA/HFS are presented in Tables S7 and S8. Interestingly, several common GO terms related to immunity were over-represented after these treatments but never found after HFS alone. From the eight genes significantly altered in both DOPA and DOPA/HFS groups, three genes were involved in immune responses: *C1s* and *Rt1-Da* were up-regulated and *Irf7* was down-regulated.

Two KEGG metabolic pathways involved in the striatal effect of HFS were identified (Table 2). The first one, “calcium signaling pathway”, grouped together 5 genes among which *Chrna7*, encoding the α7-nicotinic acetylcholine receptor, which has been notably involved in the neuroprotective action of nicotine for dopamine neurons. The second one is “type II diabetes mellitus”; interestingly, there are reports suggesting a link between this pathology and PD [28]. Three KEGG pathways were identified after L-DOPA treatment: “neuroactive ligand-receptor interaction”, “calcium signaling pathway”, and “ECM-receptor interaction”. Some of the genes from these pathways are related to non-dopaminergic transmitter systems that have been implicated in LIDs, such as *Adra2b* and *Adrb1* (encoding adrenergic alpha 2 and beta1 receptors), *Htr2c* (5-hydroxytryptamine/serotonin receptor 2C), *Cnr1* (cannabinoid receptor 1 or CB1). Several genes in the calcium pathway underwent similar down-regulation under HFS and DOPA, suggesting that their regulation might be associated rather to the beneficial effects of these treatments than to LIDs. The genes clustered in the “ECM-receptor interaction” pathway include *Sdc1* (syndecan 1) and genes encoding integrins and type V collagen. Only one KEGG pathway, “calcium signaling pathway”, was found when testing the genes differentially regulated in the striatum of rats after DOPA/HFS (Table 2). This pathway was thus common to the 3 treatment groups although the genes regulated were mostly not the same, except *Adrb1* which was similarly regulated in the 3 treatment conditions.

**Table 2:** KEGG metabolic pathways for genes differentially regulated in HFS, DOPA and DOPA/HFS groups vs 6-OHDA.

*Gene symbol*	Gene name
**HFS vs 6-OHDA**	
**Calcium signaling pathway, p value 0.026**	
*Adrb1*	adrenergic, beta-1-, receptor
*Cacna1b*	calcium channel, voltage-dependent, N type, alpha 1B subunit
*Chrna7*	cholinergic receptor, nicotinic, alpha 7
*Itpka*	inositol 1,4,5-trisphosphate 3-kinase A
*Prkcb*	protein kinase C, beta
**Type II diabetes mellitus, p value 0.037**	
*Cacna1b*	calcium channel, voltage-dependent, N type, alpha 1B subunit
*Mapk8*	mitogen-activated protein kinase 8
*Prkcd*	protein kinase C, delta
**DOPA vs 6-OHDA**	
**Neuroactive ligand-receptor interaction, p value 0.02**	
*Htr2c*	5-hydroxytryptamine (serotonin) receptor 2C
*Mas1*	MAS1 oncogene
*Adra2b*	adrenergic, alpha-2B-, receptor
*Adrb1*	adrenergic, beta-1-, receptor
*Agtr1a*	angiotensin II receptor, type 1a
*Cnr1*	cannabinoid receptor 1 (brain)
*Crhr1*	corticotropin releasing hormone receptor 1
*Grm4*	glutamate receptor, metabotropic 4
*Hcrtr1*	hypocretin (orexin) receptor 1
**ECM-receptor interaction, p value 0.02**	
*Col5a2*	collagen, type V, alpha 2
*Col5a3*	collagen, type V, alpha 3
*Itga7*	integrin alpha 7
*Itgb6*	integrin, beta 6
*Sdc1*	syndecan 1
**Calcium signaling pathway, p value 0.03**	
*Htr2c*	5-hydroxytryptamine (serotonin) receptor 2C
*ATP2B1*	ATPase, Ca++ transporting, plasma membrane 1
*Adrb1*	adrenergic, beta-1-, receptor
*Agtr1a*	angiotensin II receptor, type 1a
*Itpka*	inositol 1,4,5-trisphosphate 3-kinase A
*Prkcb*	protein kinase C, beta
*Ppp3r1*	protein phosphatase 3, regulatory subunit B, alpha isoform (calcineurin B, type I)
**DOPA/HFS vs 6-OHDA**	
**Calcium signaling pathway, p value 0.05**	
*Adrb1*	adrenergic, beta-1-, receptor
*Chrm3*	cholinergic receptor, muscarinic 3
*Tnnc2*	troponin C type 2 (fast)

## Discussion

### Molecular mechanisms underlying the action of subchronic STN-HFS

Several genes involved in neuroplasticity or neuroprotection were altered under STN-HFS (Table 3).

**Table 3:** Functional implications of the products of the genes differentially expressed after STN-HFS in link with neuroplasticity, neurogenesis or neuroprotection.

NEUROPLASTICITY/NEUROGENESIS			
**Increased by STN-HFS, localization: extracellular**			
TTR transthyretin	Transport of retinol	Retinol regulates striatal neuron differentiation and adult neurogenesis (post stroke striatal adult neurogenesis)	[31], [32], [33]
IGF2	Growth factor	Involved in dopaminergic differentiation, adult neurogenesis; modulator of spine morphology	[44], [45]
SOSTDC1	BMP antagonist	BMP antagonists are involved in differentiation/recruitment of neurons in the striatum	[48], [49]
**Increased by STN-HFS, localization: nuclear**			
Nr4A3	Transcription factor	NR4A gene expression is related to expression of dopamine neurotransmission genes.	[52]
**Decreased by STN-HFS, localization: nuclear**			
Ebf1	Transcription factor	Involved in differentiation of striatonigral neurons	[54]

NEUROPROTECTION			
**Increased by STN-HFS, localization: membrane or extracellular**			
CHRNA7	Binds Acholine/nicotine	Involved in the neuroprotective effect of nicotine on DA neurons; Increases IGF2	[65], [67]
TTR	Binds Aβ, Metallopeptidase	Prevents Aβ amyloid fibril formation Degrades ApoA-1, NPY, Aβ	[29], [34]
**Decreased by STN-HFS, localization: intracellular**			
PRKCd	Protein kinase	PRKCd inhibition prevents dopaminergic neurodegeneration	[59], [60]
BBC3	Pro-apoptotic protein	Involved in 6-OHDA-induced cell death	[62]
SIRT5	NAD-dependent protein deacetylase	Promotes neuronal death	[64]

#### STN-HFS and plasticity-associated genes

A main finding from this study is the drastic increase in *Ttr* expression induced specifically by STN-HFS. Transthyretin (TTR) is mostly known as a carrier of thyroxin and indirectly of retinol via binding to the retinol-binding protein. Until recently, the only site of TTR synthesis in the brain was thought to be the choroid plexus, but evidence has been provided that neurons transcribe TTR mRNA [29]. Decreased TTR expression during aging has been associated with reduced availability of the retinol-derived retinoic acid (RA) and with memory deficit [30]. RA is an important morphogen in brain development, especially in striatal neuron differentiation [31], and a key regulator of neuroplasticity in adult brain, including normal adult neurogenesis and post-stroke striatal neurogenesis [32], [33]. It is therefore of interest to associate the *Ttr* upregulation shown here with our previous finding that similar subchronic STN-HFS promotes newly-formed cell survival, not only in the regions of constitutive neurogenesis, but also in the striatum [13]. Recently, TTR has been shown to act as a metallopeptidase [34] with 3 natural substrates identified, apoA-1, neuropeptide Y and Aβ. Interestingly, neuropeptide Y is expressed in a population of striatal GABA interneurons [35] and is upregulated in the striatum in PD patient [36] and rat PD model [37], [38]. By preventing the formation of amyloid beta fibrils, TTR may also play a neuroprotective role in degenerative diseases such as Alzheimer disease [29]. In connection with PD, *Ttr* is upregulated at a pre-manifest stage in the SNc of MPTP-treated monkeys [39] and in the striatum of mice overexpressing human α-synuclein gene [40], suggesting a role in compensatory processes. *Ttr* is also upregulated in response to nicotine [41], which, according to epidemiological data and studies in animal models, may protect against PD [42]. Based on these data, the increase in striatal *Ttr* gene expression found here under subchronic STN-HFS might be part of a molecular pathway underlying neuroprotective/neuroadaptative effects of this surgical treatment.

Several other genes, involved in neuroplasticity are shown here to be also upregulated by STN-HFS: *Igf2*, *Sostdc1* and *Nr4a3*. Among these, only *Nr4a3* is also upregulated by L-DOPA. *Igf2* encodes the secreted growth factor IGF2 that has been suggested to have neurotrophic effects, promoting survival and differentiation of neuronal cell [43], to modulate neuronal plasticity and to regulate adult neurogenesis in the hippocampus [44]. Interestingly, IGF2 has been pointed as part of a combination of 4 factors sufficient to promote differentiation of human embryonic stem cells to functional midbrain DA neurons [45]. Moreover, IGF2 was recently identified as a potent modulator of synapse density and spine morphology in mature hippocampal neurons [46]. **S**triatal spine loss is a key pathological feature of Parkinson's disease [47]. Whether or not STN-HFS might restore synaptic architecture at spines through IGF2-mediated mechanisms thus deserves particular interest for future investigations.

The protein SOSTDC1 acts as a bone morphogenetic protein (BMP) antagonist, as does NOGGIN that has been shown to suppress the BMP-mediated pro-gliogenic signaling and promote the differentiation/recruitment of neurons in the adult striatum [48], [49].

*Nr4a3* is upregulated by STN-HFS and L-DOPA treatments, with a prominent increase under HFS. NR4A3 is a member of the NR4A family of nuclear receptors [50], which depending on their level of expression and subcellular localization are involved in differentiation, survival and apoptosis. Induction of *Nr4a3* gene has been associated to neuroleptic-mediated striatal responses [51] and suggested to regulate tyrosine hydroxylase and dopamine transporter DAT [52] suggesting a role in DA transmission at post- and pre-synaptic level.

Another gene of interest specifically regulated by STN-HFS is *Ebf1*. *Ebf1* is one of the 2 genes commonly down-regulated in the HFS and DOPA-HFS conditions. It is a member of a family of transcription factors implicated in the coupling of neuronal differentiation with cell cycle exit and possibly stabilization of the committed state [53]. EBF1 has been demonstrated to be a lineage-specific transcription factor critical to the differentiation of striatonigral neurons [54] that form the D1 receptor-expressing direct pathway. Imbalanced activity between this pathway and the D2R-expressing indirect pathway is an essential pathophysiological feature of PD. How is the down-regulation of *Ebf1* by STN-HFS impacting the striatonigral pathway is an intriguing issue that would deserve further investigation.

#### STN-HFS and genes associated to cell death or neuroprotection

Several genes possibly involved in cell death/apoptosis are down-regulated by STN-HFS *Prkcd*, *Bbc3*, *Sirt5*, whereas *Chrna7* presumably involved in neuroprotection is upregulated. Modulation of these genes may then be part of striatal adaptive processes promoting survival of newly-formed striatal cells [13], protecting striatal neurons, or contributing, at axon terminal level, to the previously reported neuroprotective effects of STN surgery on nigral DA neurons [51]–[54] and to increased metabolism of spared DA neuron [55]–[57].

PRKCD transcriptional upregulation is implicated in oxidative stress-induced neuronal cell death [58]. Conversely, pharmacological inhibition of PRKCD or depletion by siRNA is sufficient to prevent dopaminergic neurodegeneration in PD models [59], [60] pointing to PRKCD inhibition as a neuroprotective strategy for PD. It is to note that *Prkcd* is one of the two genes oppositely regulated by STN-HFS and L-DOPA; its upregulation by L-DOPA complies with previous report [14] and supports pro-oxidant property of L-DOPA at high dosage inducing dyskinesia.

BBC3 is a pro-apoptotic protein, acting as main regulator of oxidative stress and neuronal apoptosis and has been proposed as a target for the treatment of neurodegenerative conditions [61]. In connection to PD, induction of *Bbc3* has been involved in 6-OHDA-induced cell death mechanisms [62]. *Sirt5*, encodes a mitochondrial isoform of sirtuins, a family of NAD+-dependent protein deacetylases [63]. Growing evidence suggests that sirtuins are involved in neuronal survival, with however opposite effects: for instance, whereas Sirt1 is generally considered as neuroprotective, a recent report shows that mitochondrial Sirt5 can promote neuronal death [64]. Finally, our study evidences STN-HFS-induced upregulation of α7-nicotinic acetylcholine receptor gene (*Chrna7*). In addition to its role in the modulation of specific neurotransmitters and as a central regulator of inflammatory processes, CHRNA7 (α7 nAChR) has been involved in neuroprotection following various insults. In particular, it has been reported to mediate the neuroprotective effect of nicotine on dopaminergic neurons [65]. These receptors are expressed in a high percentage of the striatal cholinergic interneuron population [66], the only known source of acetylcholine in the striatum. Also few in numbers, these interneurons are important regulators of striatal function. Interestingly, activation of α7 nAChRs has been shown in hippocampus to increase Igf2 expression [67], and nicotine, as reported above, to increase Ttr expression, suggesting a link between up-regulation of these 3 genes in a neuroprotective mechanism.

Finally, STN-HFS also upregulates *Ngfr* and down-regulates *Btg2 and Carhsp1. Ngfr* encodes the p75 neurotrophin receptor (p75^NTR^) and has been involved in both pro-apoptotic and prosurvival processes through multiple signaling cascades depending on the cellular context [68]. *Btg2* has been identified as part of a set of activity-regulated inhibitor of death genes that mediates acquired neuroprotection induced by synaptic activity, in particular pro-survival effect through synaptic NMDA receptors [69]. As corticostriatal glutamate transmission is markedly increased after dopamine lesion, it could be that this gene is upregulated as a neuroprotection mechanism against excitotoxicity in PD state and that its down-regulation by STN-HFS vs 6-OHDA found here is linked to the previously reported suppression by STN-HFS of overactive striatal glutamate transmission [9]. *Carhsp1* is one of the two genes commonly regulated in HFS and DOPA-HFS conditions but not by DOPA alone. It encodes a cold-shock domain-containing protein which has been recently identified as a TNF-α mRNA stability enhancer required for effective production of TNF- α, a central mediator of inflammation [70]. Carhsp1 down-regulation by STN-HFS might then also participate to neuroprotection by reducing inflammation.

### Genes not regulated by HFS potentially involved in L-DOPA-induced dyskinesia

L-DOPA at a dose inducing dyskinesia is shown here to alter numerous genes in the striatum, the great majority of which were not modified by HFS alone applied with parameters efficiently alleviating akinesia, without inducing dyskinesias. The upregulation of *Pdyn* observed here is a consistent correlate of LIDs across species and parkinsonian conditions [22], [71]–[73] and is in accordance with an over-activation of the direct striatal pathway. Several modulated genes could contribute to the abnormal glutamate transmission that has long been proposed to play a role in LID [74], [75]. This includes the decrease in *Grm4* that encodes metabotropic glutamate receptor 4 (mGlu4) and the down-regulation of Ppp3r1, the calcineurin regulatory unit gene, knowing that calcineurin function is closely related to NMDA-receptor signaling and involved in dopamine-glutamate interactions. In accordance with previous reports [14], [76], gene expression of type 1 cannabinoid receptor (CB1), considered as a target for antidyskinetic therapies, is upregulated. The largest and most significant change induced by L-DOPA reported here and in accordance with previous studies [15], [77] is the upregulation of *Trh*. This could be an essential feature of dyskinesias because the intrastriatal injection of TRH induces abnormal movements, apparently by increasing dopamine release. Finally, LIDs have been shown to also involve the noradrenergic and serotonin systems [78]. In this connection, we evidence a down-regulation of striatal adrenergic alpha2 (*α2bR* or *Adra2b*) and serotonin 2C (Htr2c or 5-*Ht2c*) receptor genes. Although these receptors have been pointed as potential targets for LIDs [5], [79], the direct link between their down-regulation at striatal level found here and LIDs mechanisms remains elusive. It is to note that several other genes reported here have been already identified in previous microarray studies on LIDs: *Atp2b1*, *Arbp, Cdh22, Igfbp5, Itpka, Itga7, Nefh, Ppp3ca, Prkcd, Rgc32, Rgs4, Scg2, Tnni3* [14]–[18]. Differentially expressed genes in the DOPA group also include genes clustered in the KEGG pathway ECM-receptor interaction, such as syndecan 1, a transmenbrane heparansulfate proteoglycan, which has been notably involved in angiogenesis and in astrocytic reactivity in response to brain injury. These genes could contribute to LID-associated reorganizations, such as the previously reported microvascular remodeling [80].

In our experiments, HFS exacerbates LIDs when applied with a dyskinesiogenic L-DOPA treatment. We identified three novel genes possibly associated with LIDs as they are commonly modified in the DOPA and DOPA/HFS groups but unaffected by HFS alone. These genes are involved in immune response: *C1s* and *Rt1*-*Da* are up-regulated and *Irf7* is down-regulated. Several GO terms related to immunity are also found in common in L-DOPA and L-DOPA/HFS and never found after HFS alone. Increasing evidence support the involvement of innate and adaptive immunity in PD progression [81]; altered immune system in PD might in part be linked to an immunoregulatory role of dopamine [82]. Whether the expression of LIDs might involve an abnormal immune response then deserves further investigation. On the other hand, a number of genes (15) are regulated only in the DOPA/HFS condition and one cannot exclude their contribution to the reinforcement of LIDs by STN-HFS. These genes are quite all down-regulated and include the muscarinic M3 receptor and a subunit of the hyperpolarization-activated cyclic nucleotide-gated potassium channels (HCN) that are responsible for the i(h) current characterizing the cholinergic interneurons in the striatum and underlying their tonic activity. Interestingly, recent data have suggested that cholinergic neurons may play a central role upstream of striatal projection neurons once LID is established [83].

### Genes commonly regulated by HFS and/or L-DOPA

The KEGG pathway “Calcium signaling pathway” is the only one to be associated with the 3 treatment conditions. However, there is a single gene commonly regulated in this pathway: *Adrb1*, which encodes the adrenergic β1-receptor and which is down-regulated. This finding supports a major role of these receptors in PD pathophysiology. These receptors are highly expressed in the striatum where their activation results in excitation of cholinergic interneurons and medium spiny neurons through cAMP-dependent pathway [84], [85]. Their blockade has been suggested to mediate the beneficial effects of propranolol on PD tremor and on LIDs. Outside this KEGG pathway, there are few (4) other genes commonly regulated under the 3 treatment conditions, among which *btg2*, *Sirt5* and *Ngfr* that have been involved in cell death or neuroprotection. These commonly regulated genes may thus represent molecular targets of interest for antiparkinsonian therapies.

In conclusion, this study provides evidence that STN-HFS in hemiparkinsonian rats induces widespread molecular changes in the striatum, suggesting important anatomo-functional remodeling, in particular expression of genes involved in plasticity/neuroprotection pathways that might create an environment favorable for protection/sprouting of DA terminals or adult neurogenesis. Our data also point to genes of the immune system as potentially involved in LIDs and to adrenergic β1-receptor as a common player of antiparkinsonian therapies.

## Supporting Information

Table S1 **Primer sequences used for quantitative real time RT-PCR.**
(DOCX)

Table S2 **Global gene ANOVA analysis.**
(DOCX)

Table S3 **Genes differentially regulated in the striatum of rats after HFS demonstrated by microarray analysis.** Two class unpaired Significance Analysis of Microarrays (SAM) of TMev with 20 permutations and a 6% FDR was used to analyze the microarray data of striatal gene expression in the HFS vs 6-OHDA groups: fold changes with values higher than 1 indicate up-regulation of gene expression after HFS and fold changes with values less than 1 indicate down-regulation of expression.
(DOCX)

Table S4 **Genes differentially expressed in the striatum of rats after L-DOPA treatment demonstrated by microarray analysis.** Two class unpaired Significance Analysis of Microarrays (SAM) of TMev with 1% FDR was used to analyze the microarray data of striatal gene expression in the DOPA vs 6-OHDA group: fold changes with values higher than 1 indicate up-regulation of gene expression after HFS and fold changes with values less than 1 indicate down-regulation of expression.
(DOCX)

Table S5 **Genes differentially regulated in the striatum of rats after combined L-DOPA and HFS treatment demonstrated by microarray analysis.** Two class unpaired Significance Analysis of Microarrays (SAM) of TMev with a 12% FDR was used to analyze the microarray data of striatal gene expression in the DOPA/HFS vs 6-OHDA groups: fold changes with values higher than 1 indicate up-regulation of gene expression after HFS and fold changes with values less than 1 indicate down-regulation of expression.
(DOCX)

Table S6 **Functional annotation chart: Most relevant biological terms associated with HFS.** Abbreviations: GO: gene ontology; BP: biological process; CC: cell. Component; MF: molecular function.
(DOCX)

Table S7 **Functional annotation chart: Most relevant biological terms associated with DOPA.**
(DOCX)

Table S8 **Functional annotation chart: Most relevant biological terms associated with DOPA/HFS.**
(DOCX)
