# Supplementary material for: Striatal Molecular Signature of Subchronic Subthalamic Nucleus High Frequency Stimulation in Parkinsonian Rat
Source: PLoS One. 2013 Apr 4;8(4):e60447. doi: 10.1371/journal.pone.0060447 (PMC3617149; doi:10.1371/journal.pone.0060447)
Supplement: Table S1 — Primer sequences used for quantitative real time RT-PCR. (DOCX) [file pone.0060447.s001.docx]

Table S1. Primer sequences used for quantitative real time RT-PCR

| **Gene**  **name** | **Genbank ID** | Primer sequence |
| --- | --- | --- |
| Ca4 | NM_019174.1 | F: CGT CTATGCCCTCAAGCA C  R: TCTCATAGCACCAGTGTGAATCTT |
| Igf2 | NM_031511.1 | F: CGCTTCAGTTTGTCTGTTCG  R: GCAGCACTCTTCCACGATG |
| Chrna7 | NM_012832.3 | F: GGCAAAATGCCTAAGTGGAC  R: CTTCATGCGCAGAAACCA T |
| Hprt1 | NM_012583.2 | F: GACCGGTTCTGTCATGTCG  R: ACCTGGTTCATCATCACTAATCAC |
| PrKcd | NM_133307.1 | F: CCCTTTAAGCCCAAAGTGAA  R: TTGGGGTTTCTCATTCAGGA |
| TTr | NM_012681.1 | F: GCCTCGCTGGACTGATATTT  R: GACACTTGGATTCTCCAGCAC |
| CLIC6 | NM_176078.2 | F: GAGCACGACATCACCCTCTT  R: GCAGTTTCCGATGCTCTCTC |
| Sostdc1 | NM_153737.1 | F: CAGCAACAGCACCTTGAATC  R: CAGCCCACTTGAACTCGACTA |
| Sirt5 | NM_001004256.1 | F: GGAGAAAATGGCAAGCTCAG  R: GTGAGGGGTTGTGAGCAAAG |
