# Supplementary material for: Striatal Molecular Signature of Subchronic Subthalamic Nucleus High Frequency Stimulation in Parkinsonian Rat
Source: PLoS One. 2013 Apr 4;8(4):e60447. doi: 10.1371/journal.pone.0060447 (PMC3617149; doi:10.1371/journal.pone.0060447)
Supplement: Table S2 — Global gene ANOVA analysis. (DOCX) [file pone.0060447.s002.docx]

Table S2: Global gene ANOVA analysis

| Factor | Sum of the squares | DOF | Variance | F | P-value |
| --- | --- | --- | --- | --- | --- |
| Gene | 1654381.04 | 26459 | 62.53 | 460.66 | <10^-5^ |
| DOPA | 79.96 | 1 | 79.96 | 589.07 | <10^-5^ |
| HFS | 64.5 | 1 | 64.5 | 475.18 | <10^-5^ |
| DOPA/HFS | 86.58 | 1 | 86.58 | 637.86 | <10^-5^ |
| Residual | 28731.76 | 211680 | 0.14 | - | - |
