# Supplementary material for: Striatal Molecular Signature of Subchronic Subthalamic Nucleus High Frequency Stimulation in Parkinsonian Rat
Source: PLoS One. 2013 Apr 4;8(4):e60447. doi: 10.1371/journal.pone.0060447 (PMC3617149; doi:10.1371/journal.pone.0060447)
Supplement: Table S3 — Genes differentially regulated in the striatum of rats after HFS demonstrated by microarray analysis. Two class unpaired Significance Analysis of Microarrays (SAM) of TMev with 20 permutations and a 6% FDR was used to analyze the microarray data of striatal gene expression in the HFS vs 6-OHDA groups: fold changes with values higher than 1 indicate up-regulation of gene expression after HFS and fold changes with values less than 1 indicate down-regulation of expression. (DOCX) [file pone.0060447.s003.docx]

Table S3: Genes differentially regulated in the striatum of rats after HFS demonstrated by microarray analysis.

| **GENE_SYMBOL** | **Gene Name** | **Fold change** |
| --- | --- | --- |
| Agt | angiotensinogen (serpin peptidase inhibitor. clade A. member 8) | 1.30 |
| Aqp1 | aquaporin 1 | 3.27 |
| Abcd2 | ATP-binding cassette. sub-family D (ALD). member 2 | 0.76 |
| Adrb1 | adrenergic. beta-1-. receptor | 0.74 |
| Aph1a | anterior pharynx defective 1 homolog A (C. elegans); similar to Gamma-secretase subunit APH-1A (APH-1a) (Aph-1alpha) (Presenilin-stabilization factor) | 0.75 |
| Bbc3 | Bcl-2 binding component 3 | 0.51 |
| Bcat2 | branched chain aminotransferase 2. mitochondrial | 0.78 |
| Bhlhe40 | basic helix-loop-helix family. member e40 | 0.55 |
| Btg2 | B-cell translocation gene 2. anti-proliferative | 0.62 |
| C1qtnf6 | C1q and tumor necrosis factor related protein 6 | 0.70 |
| Ca4 | anhydrase carbonique IV | 0.61 |
| Cacnb2 | calcium channel. voltage-dependent. beta 2 subunit | 0.75 |
| Cacna1b | calcium channel. voltage-dependent. N type. alpha 1B subunit | 0.76 |
| Carhsp1 | calcium regulated heat stable protein 1 | 0.80 |
| Chrna7 | cholinergic receptor. nicotinic. alpha 7 | 1.34 |
| Ddhd1 | DDHD domain containing 1 | 0.77 |
| Dll1 | delta-like 1 (Drosophila) | 0.79 |
| Dnajb9 | DnaJ (Hsp40) homolog. subfamily B. member 9 | 0.72 |
| Ebf1 | early B-cell factor 1 | 0.68 |
| Edn1 | endothelin 1 | 0.74 |
| Emp3 | epithelial membrane protein 3 | 0.73 |
| Enpp1 | ectonucleotide pyrophosphatase/phosphodiesterase 1 | 0.79 |
| Entpd5 | ectonucleoside triphosphate diphosphohydrolase 5 | 0.70 |
| Foxq1 | forkhead box Q1 | 0.76 |
| Galntl5 | UDP-N-acetyl-alpha-D-galactosamine:polypeptide N-acetylgalactosaminyltransferase-like 5 | 1.80 |
| Geft | RhoA/RAC/CDC42 exchange factor | 0.74 |
| Gnb1 | guanine nucleotide binding protein (G protein). beta polypeptide 1; guanine nucleotide binding protein (G protein). beta polypeptide 4 | 0.72 |
| Gpc3 | glypican 3 | 0.73 |
| Gstm5 | glutathione S-transferase. mu 5 | 1.25 |
| Gtpbp8 | GTP-binding protein 8 (putative) | 0.69 |
| Hsd3b7 | hydroxy-delta-5-steroid dehydrogenase. 3 beta- and steroid delta-isomerase 7 | 0.79 |
| Htra1 | HtrA serine peptidase 1 | 1.36 |
| Igf2 | insulin-like growth factor 2 | 2.04 |
| ItpkA | inositol 1.4.5-trisphosphate 3-kinase A | 0.77 |
| Klc3 | kinesin light chain 3 | 1.42 |
| Lamp2 | lysosomal-associated membrane protein 2 | 1.32 |
| LOC314600 | similar to zinc finger protein 422. related sequence 1 | 0.72 |
| LOC361990 | similar to DKFZP547E1010 protein; similar to Protein C1orf77 homolog | 1.37 |
| LOC378467 | promethin | 0.69 |
| LOC683470 | similar to growth arrest specific 1 | 0.70 |
| LOC684626 | similar to K11B4.2 | 0.60 |
| LOC688966 | MEF2B neighbor | 0.40 |
| Mapk8 | mitogen-activated protein kinase 8 | 1.20 |
| Nfil3 | nuclear factor. interleukin 3 regulated | 1.46 |
| NHLrc1 | NHL repeat containing 1 | 0.76 |
| Ngfr | nerve growth factor receptor (TNFR superfamily. member 16) | 1.27 |
| Nr4a3 | nuclear receptor subfamily 4. group A. member 3 | 2.70 |
| Pars2 | prolyl-tRNA synthetase 2. mitochondrial (putative) | 0.77 |
| Pdia3 | protein disulfide isomerase family A. member 3 | 1.48 |
| Prelp | proline arginine-rich end leucine-rich repeat protein | 1.24 |
| Prkcb | protein kinase C. beta | 0.76 |
| Prkcd | protein kinase C. delta | 0.61 |
| Rab3Gap2 | RAB3 GTPase activating protein subunit 2 | 0.61 |
| RGD1310039 | similar to hypothetical protein FLJ10058 | 1.25 |
| Rpe65 | retinal pigment epithelium 65 | 1.40 |
| Serpina4 | serine (or cysteine) proteinase inhibitor. clade A (alpha-1 antiproteinase. antitrypsin). member 4 | 0.80 |
| Sirt5 | sirtuin (silent mating type information regulation 2 homolog) 5 (S. cerevisiae) | 0.44 |
| Slc18A2 | solute carrier family 18 (vesicular monoamine). member 2 | 0.77 |
| Slc39A8 | solute carrier family 39 (metal ion transporter). member 8 | 0.76 |
| Slc4A2 | solute carrier family 4 (anion exchanger). member 2 | 1.46 |
| Slc12a4 | solute carrier family 12 (potassium/chloride transporters). member 4 | 1.24 |
| Sostdc1 | sclerostin domain containing 1 | 25.91 |
| Tcirg1 | T-cell. immune regulator 1. ATPase. H+ transporting. lysosomal V0 subunit A3 | 0.79 |
| Timm8B | translocase of inner mitochondrial membrane 8 homolog b (yeast) | 1.28 |
| Tnfrsf11b | tumor necrosis factor receptor superfamily. member 11b | 0.75 |
| Tnfsf13 | tumor necrosis factor (ligand) superfamily. member 13 | 1.20 |
| Tradd | TNFRSF1A-associated via death domain | 0.78 |
| Trpc4 | transient receptor potential cation channel. subfamily C. member 4 | 0.78 |
| Ttr | transthyretin | 278.66 |
| Vtn | vitronectin | 0.75 |
| Wasl | IQ motif and ubiquitin domain containing; Wiskott-Aldrich syndrome-like | 1.77 |

Two class unpaired Significance Analysis of Microarrays (SAM) of TMev with 20 permutations and a 6% FDR was used to analyze the microarray data of striatal gene expression in the HFS vs 6-OHDA groups: fold changes with values higher than 1 indicate up-regulation of gene expression after HFS and fold changes with values less than 1 indicate down-regulation of expression.
