# Supplementary material for: Striatal Molecular Signature of Subchronic Subthalamic Nucleus High Frequency Stimulation in Parkinsonian Rat
Source: PLoS One. 2013 Apr 4;8(4):e60447. doi: 10.1371/journal.pone.0060447 (PMC3617149; doi:10.1371/journal.pone.0060447)
Supplement: Table S4 — Genes differentially expressed in the striatum of rats after L-DOPA treatment demonstrated by microarray analysis. Two class unpaired Significance Analysis of Microarrays (SAM) of TMev with 1% FDR was used to analyze the microarray data of striatal gene expression in the DOPA vs 6-OHDA group: fold changes with values higher than 1 indicate up-regulation of gene expression after HFS and fold changes with values less than 1 indicate down-regulation of expression. (DOCX) [file pone.0060447.s004.docx]

Table S4: Genes differentially expressed in the striatum of rats after L-DOPA treatment demonstrated by microarray analysis.

| **GENE_SYMBOL** | **Gene Name** | **Fold change** |
| --- | --- | --- |
| Adra2b | adrenergic. alpha-2B-. receptor | 0.79 |
| Adrb1 | adrenergic. beta-1-. receptor | 0.66 |
| AgmaT | agmatine ureohydrolase (agmatinase) | 0.78 |
| Agtr1a | angiotensin II receptor. type 1a | 0.77 |
| Aldh1a1 | aldehyde dehydrogenase 1 family. member A1 | 1.27 |
| Amacr | alpha-methylacyl-CoA racemase | 0.76 |
| Amhr2 | anti-Mullerian hormone receptor. type II | 1.99 |
| Anp32A | acidic (leucine-rich) nuclear phosphoprotein 32 family. member A | 1.71 |
| Anxa7 | similar to Annexin A7 (Annexin VII) (Synexin); annexin A7 | 0.77 |
| Arbp | acidic ribosomal phosphoprotein P0 | 0.79 |
| Arhgap24 | Rho GTPase activating protein 24 | 1.25 |
| Atf3 | activating transcription factor 3 | 0.68 |
| ATP2b1 | ATPase. Ca++ transporting. plasma membrane 1 | 1.22 |
| Banf1 | similar to barrier to autointegration factor 1; barrier to autointegration factor 1 | 0.79 |
| Bbc3 | Bcl-2 binding component 3 | 0.66 |
| Bcap31 | B-cell receptor-associated protein 31 | 1.30 |
| Bex4 | brain expressed gene 4 | 0.71 |
| Btg2 | B-cell translocation gene 2. anti-proliferative | 0.56 |
| Bub1b | budding uninhibited by benzimidazoles 1 homolog. beta (S. cerevisiae) | 0.70 |
| C1s | similar to complement component 1. s subcomponent; complement component 1. s subcomponent | 1.32 |
| Camkk1 | calcium/calmodulin-dependent protein kinase kinase 1. alpha | 0.72 |
| Cand2 | cullin-associated and neddylation-dissociated 2 (putative) | 0.77 |
| Cd74 | Cd74 molecule. major histocompatibility complex. class II invariant chain | 1.81 |
| Cdh22 | cadherin 22 | 1.57 |
| Cds1 | CDP-diacylglycerol synthase 1 | 1.30 |
| Cebpb | CCAAT/enhancer binding protein (C/EBP). beta | 0.77 |
| Cflar | CASP8 and FADD-like apoptosis regulator | 1.67 |
| Cgref1 | cell growth regulator with EF hand domain 1 | 1.40 |
| Ch25h | cholesterol 25-hydroxylase | 0.57 |
| Cited4 | Cbp/p300-interacting transactivator. with Glu/Asp-rich carboxy-terminal domain. 4 | 0.67 |
| Clcnkb | chloride channel Kb | 1.35 |
| Cldn5 | claudin 5 | 1.22 |
| Cnr1 | cannabinoid receptor 1 (brain) | 1.79 |
| Cntn2 | contactin 2 (axonal) | 0.73 |
| Cntn6 | contactin 6 | 1.22 |
| Col5a2 | collagen. type V. alpha 2 | 1.48 |
| Col5a3 | collagen. type V. alpha 3 | 0.76 |
| Cpne9 | copine family member IX | 2.22 |
| Crabp2 | cellular retinoic acid binding protein 2 | 1.55 |
| Crhr1 | corticotropin releasing hormone receptor 1 | 2.05 |
| Cryba2 | crystallin. beta A2 | 2.08 |
| Csf1 | colony stimulating factor 1 (macrophage) | 0.76 |
| Cspg4 | chondroitin sulfate proteoglycan 4 | 0.78 |
| Ctsk | cathepsin K | 2.23 |
| Dcx | doublecortin | 0.66 |
| Ddit4l | DNA-damage-inducible transcript 4-like | 0.79 |
| Dhfr | similar to dihydrofolate reductase; dihydrofolate reductase | 0.76 |
| Dhrs3 | dehydrogenase/reductase (SDR family) member 3 | 1.40 |
| Dnase2b | deoxyribonuclease II beta | 1.22 |
| Efemp2 | similar to epidermal growth factor-containing fibulin-like extracellular matrix protein 2; EGF-containing fibulin-like extracellular matrix protein 2 | 2.32 |
| Egln3 | EGL nine homolog 3 (C. elegans) | 0.76 |
| Emp3 | epithelial membrane protein 3 | 1.45 |
| Fbl | fibrillarin | 0.80 |
| Filipi | filamin A interacting protein 1 | 0.79 |
| Fos | FBJ osteosarcoma oncogene | 0.46 |
| Fzd1 | frizzled homolog 1 (Drosophila) | 0.67 |
| Kcnj13 | potassium channel. subfamily K. member 13 | 2.27 |
| Gadd45A | growth arrest and DNA-damage-inducible. alpha | 1.38 |
| Galntl5 | UDP-N-acetyl-alpha-D-galactosamine:polypeptide N-acetylgalactosaminyltransferase-like 5 | 1.45 |
| Gap43 | growth associated protein 43 | 0.79 |
| Geft | RhoA/RAC/CDC42 exchange factor | 0.74 |
| Gnrh1 | gonadotropin-releasing hormone 1 (luteinizing-releasing hormone) | 0.71 |
| Gpr153 | G protein-coupled receptor 153 | 1.47 |
| Gpr26 | G protein-coupled receptor 26 | 1.81 |
| Grem1 | gremlin 1. cysteine knot superfamily. homolog (Xenopus laevis) | 1.61 |
| Grm4 | glutamate receptor. metabotropic 4 | 0.74 |
| Gss | glutathione synthetase | 1.47 |
| Gucy1b3 | guanylate cyclase 1. soluble. beta 3 | 0.79 |
| Hal | histidine ammonia lyase | 0.77 |
| Hcrtr1 | hypocretin (orexin) receptor 1 | 1.72 |
| Hes3 | hairy and enhancer of split 3 (Drosophila) | 1.51 |
| Hpgd | hydroxyprostaglandin dehydrogenase 15 (NAD) | 1.60 |
| Hsd17b8 | hydroxysteroid (17-beta) dehydrogenase 8 | 0.79 |
| Htr2c | 5-hydroxytryptamine (serotonin) receptor 2C | 0.78 |
| Ifitm3 | interferon induced transmembrane protein 3 | 0.69 |
| Igfbp5 | insulin-like growth factor binding protein 5 | 0.63 |
| Igsf10 | immunoglobulin superfamily. member 10 | 0.67 |
| Ilf3 | interleukin enhancer binding factor 3 | 0.76 |
| Inhba | inhibin beta-A | 1.93 |
| Inhbb | inhibin beta-B | 0.67 |
| Irf7 | interferon regulatory factor 7 | 0.66 |
| Itga7 | integrin alpha 7 | 1.56 |
| Itgb6 | integrin. beta 6 | 0.62 |
| ItpkA | inositol 1.4.5-trisphosphate 3-kinase A | 0.70 |
| Junb | jun B proto-oncogene | 0.65 |
| Kcnab3 | potassium voltage-gated channel. shaker-related subfamily. beta member 3 | 1.62 |
| Lgals3 | lectin. galactoside-binding. soluble. 3 | 2.31 |
| Lilrb3 | leukocyte immunoglobulin-like receptor. subfamily B (with TM and ITIM domains). member 3; similar to paired-Ig-like receptor A11; leukocyte immunoglobulin-like receptor. subfamily B (with TM and ITIM domains). member 3-like; paired-Ig-like receptor A2 | 0.77 |
| LOC313672 | kazrin | 1.36 |
| LOC499749 | similar to RIKEN cDNA C430004E15 | 0.80 |
| LOC503134 | hypothetical LOC503134 | 0.79 |
| LOC56825 | prochymosin | 1.87 |
| LOC683470 | similar to growth arrest specific 1 | 0.69 |
| LOC684626 | similar to K11B4.2 | 0.58 |
| LOC687813 | similar to Tnf receptor-associated factor 1 | 0.80 |
| LOC688966 | MEF2B neighbor | 0.41 |
| LOC691995 | hypothetical protein LOC691995 | 1.32 |
| Lrrc50 | leucine rich repeat containing 50 | 0.73 |
| Mas1 | MAS1 oncogene | 1.73 |
| Mrps18a | mitochondrial ribosomal protein S18A | 0.79 |
| Myh7 | myosin. heavy chain 7. cardiac muscle. beta | 0.78 |
| Nedd9 | neural precursor cell expressed. developmentally down-regulated 9 | 1.31 |
| Nefh | neurofilament. heavy polypeptide | 2.04 |
| Ngfr | nerve growth factor receptor (TNFR superfamily. member 16) | 4.58 |
| Nid1 | nidogen 1 | 0.78 |
| Nmu | neuromedin U | 2.61 |
| Nog | noggin | 0.65 |
| Npr1 | natriuretic peptide receptor 1 | 0.77 |
| Nr4a3 | nuclear receptor subfamily 4. group A. member 3 | 1.35 |
| Oas1i | 2 ' -5 ' oligoadenylate synthetase 1I | 0.65 |
| Olfm2 | olfactomedin 2 | 1.24 |
| Pafah1b3 | platelet-activating factor acetylhydrolase. isoform 1b. subunit 3 | 0.79 |
| Pdyn | prodynorphin | 2.48 |
| Pigw | phosphatidylinositol glycan anchor biosynthesis. class W | 1.23 |
| Pip5k1a | phosphatidylinositol-4-phosphate 5-kinase. type 1. alpha | 1.83 |
| Pla2g7 | phospholipase A2. group VII (platelet-activating factor acetylhydrolase. plasma) | 0.59 |
| Plekha4 | pleckstrin homology domain containing. family A (phosphoinositide binding specific) member 4 | 1.95 |
| Plunc | palate. lung. and nasal epithelium associated | 1.44 |
| Pole3 | polymerase (DNA directed). epsilon 3 (p17 subunit) | 0.74 |
| Pou3f1 | POU class 3 homeobox 1 | 0.79 |
| Ppp3r1 | protein phosphatase 3. regulatory subunit B. alpha isoform (calcineurin B. type I) | 1.34 |
| Prkcb | protein kinase C. beta | 0.72 |
| Prkcd | protein kinase C. delta | 1.69 |
| Prom2 | prominin 2 | 1.84 |
| Prp-2 | proline-rich protein | 0.76 |
| Ptpn1 | protein tyrosine phosphatase. non-receptor type 1 | 1.25 |
| Ptpn21 | protein tyrosine phosphatase. non-receptor type 21 | 0.75 |
| Rassf2 | Ras association (RalGDS/AF-6) domain family member 2 | 0.69 |
| Rbp4 | retinol binding protein 4. plasma | 1.25 |
| Rcor2 | REST corepressor 2; similar to REST corepressor 2 | 0.74 |
| Ret | ret proto-oncogene | 0.78 |
| Rexo1 | REX1. RNA exonuclease 1 homolog (S. cerevisiae) | 0.77 |
| Rexo2 | REX2. RNA exonuclease 2 homolog (S. cerevisiae) | 1.28 |
| Rgc32 | response gene to complement 32 | 1.55 |
| RGD1310352 | similar to HTGN29 protein; keratinocytes associated transmembrane protein 2 | 0.74 |
| Rgs4 | regulator of G-protein signaling 4 | 1.32 |
| Rhbdl1 | rhomboid. veinlet-like 1 (Drosophila) | 0.77 |
| Rt1-Da | histocompatibility 2. class II antigen E alpha | 1.51 |
| Samd14 | sterile alpha motif domain containing 14 | 0.63 |
| Scg2 | secretogranin II (chromogranin C) | 1.90 |
| Scn11A | sodium channel. voltage-gated. type XI. alpha | 0.80 |
| Scoc | short coiled-coil protein | 1.26 |
| Sdc1 | syndecan 1 | 1.88 |
| Sepp1 | selenoprotein P. plasma. 1 | 0.69 |
| Sfrp2 | secreted frizzled-related protein 2 | 0.78 |
| Sfxn5 | sideroflexin 5 | 2.20 |
| Sirt5 | sirtuin (silent mating type information regulation 2 homolog) 5 (S. cerevisiae) | 0.43 |
| Slc18A2 | solute carrier family 18 (vesicular monoamine). member 2 | 0.71 |
| Slc29a1 | solute carrier family 29 (nucleoside transporters). member 1 | 0.72 |
| Slc30a1 | solute carrier family 30 (zinc transporter). member 1 | 0.77 |
| Slc4A1 | solute carrier family 4 (anion exchanger). member 1 | 0.76 |
| Slc4A3 | solute carrier family 4 (anion exchanger). member 3 | 0.79 |
| Snx27 | sorting nexin family member 27 | 1.35 |
| Sqstm1 | sequestosome 1 | 0.71 |
| Stk39 | serine/threonine kinase 39. STE20/SPS1 homolog (yeast) | 1.23 |
| Sult1a1 | sulfotransferase family. cytosolic. 1A. phenol-preferring. member 1 | 1.41 |
| Syt2 | synaptotagmin II | 0.67 |
| Tagln2 | transgelin 2 | 0.80 |
| Th | tyrosine hydroxylase | 1.35 |
| Tnfsf9 | tumor necrosis factor (ligand) superfamily. member 9 | 1.49 |
| Tnni3 | troponin I type 3 (cardiac) | 1.83 |
| Tnnt1 | troponin T type 1 (skeletal. slow) | 2.66 |
| Tomm70A | translocase of outer mitochondrial membrane 70 homolog A (S. cerevisiae); similar to translocase of outer mitochondrial membrane 70 homolog A | 1.53 |
| Tpbg | trophoblast glycoprotein | 2.62 |
| Tpm1 | tropomyosin 1. alpha | 1.35 |
| Trh | thyrotropin releasing hormone | 4.77 |
| Trpc4 | transient receptor potential cation channel. subfamily C. member 4 | 0.68 |

Two class unpaired Significance Analysis of Microarrays (SAM) of TMev with 1% FDR was used to analyze the microarray data of striatal gene expression in the DOPA vs 6-OHDA group: fold changes with values higher than 1 indicate up-regulation of gene expression after HFS and fold changes with values less than 1 indicate down-regulation of expression.
