# Supplementary material for: Striatal Molecular Signature of Subchronic Subthalamic Nucleus High Frequency Stimulation in Parkinsonian Rat
Source: PLoS One. 2013 Apr 4;8(4):e60447. doi: 10.1371/journal.pone.0060447 (PMC3617149; doi:10.1371/journal.pone.0060447)
Supplement: Table S5 — Genes differentially regulated in the striatum of rats after combined L-DOPA and HFS treatment demonstrated by microarray analysis. Two class unpaired Significance Analysis of Microarrays (SAM) of TMev with a 12% FDR was used to analyze the microarray data of striatal gene expression in the DOPA/HFS vs 6-OHDA groups: fold changes with values higher than 1 indicate up-regulation of gene expression after HFS and fold changes with values less than 1 indicate down-regulation of expression. (DOCX) [file pone.0060447.s005.docx]

Table S5: Genes differentially regulated in the striatum of rats after combined L-DOPA and HFS treatment demonstrated by microarray analysis.

| **GENE_SYMBOL** | **Gene Name** | **Fold change** |
| --- | --- | --- |
| Actn1 | actinin. alpha 1 | 0.65 |
| Adrb1 | adrenergic. beta-1-. receptor | 0.79 |
| Aqp9 | aquaporin 9 | 0.55 |
| Btg2 | B-cell translocation gene 2. anti-proliferative | 0.50 |
| C1s | similar to complement component 1. s subcomponent; complement component 1. s subcomponent | 1.41 |
| Carhsp1 | calcium regulated heat stable protein 1 | 0.72 |
| Chrm3 | cholinergic receptor. muscarinic 3 | 0.53 |
| Cox6c | cytochrome c oxidase. subunit VIc | 0.73 |
| Ebf1 | early B-cell factor 1 | 0.65 |
| Egr1 | early growth response 1 | 0.65 |
| Fgfr1op2 | FGFR1 oncogene partner 2 | 0.71 |
| Galntl5 | UDP-N-acetyl-alpha-D-galactosamine:polypeptide N-acetylgalactosaminyltransferase-like 5 | 1.92 |
| Hcn2 | hyperpolarization activated cyclic nucleotide-gated potassium channel 2 | 0.79 |
| Irf7 | interferon regulatory factor 7 | 0.71 |
| Kcnh4 | potassium voltage-gated channel. subfamily H (eag-related). member 4 | 0.77 |
| LOC687516 | similar to zinc finger protein 146 | 0.72 |
| LOC691770 | similar to Ankyrin repeat domain-containing protein 28 | 0.71 |
| Mapk3 | mitogen activated protein kinase 3 | 0.62 |
| Ngfr | nerve growth factor receptor (TNFR superfamily. member 16) | 2.18 |
| Pcsk4 | proprotein convertase subtilisin/kexin type 4 | 0.71 |
| Prom1 | prominin 1 | 0.74 |
| Rt1-Da | histocompatibility 2. class II antigen E alpha | 1.71 |
| Sirt5 | sirtuin (silent mating type information regulation 2 homolog) 5 (S. cerevisiae) | 0.42 |
| Slc14A2 | solute carrier family 14 (urea transporter). member 2 | 0.78 |
| Tnnc2 | troponin C type 2 (fast) | 0.50 |
| Tsga10 | testis specific 10 | 1.49 |

Two class unpaired Significance Analysis of Microarrays (SAM) of TMev with a 12% FDR was used to analyze the microarray data of striatal gene expression in the DOPA/HFS vs 6-OHDA groups: fold changes with values higher than 1 indicate up-regulation of gene expression after HFS and fold changes with values less than 1 indicate down-regulation of expression
