# Supplementary material for: Striatal Molecular Signature of Subchronic Subthalamic Nucleus High Frequency Stimulation in Parkinsonian Rat
Source: PLoS One. 2013 Apr 4;8(4):e60447. doi: 10.1371/journal.pone.0060447 (PMC3617149; doi:10.1371/journal.pone.0060447)
Supplement: Table S6 — Functional annotation chart: Most relevant biological terms associated with HFS. Abbreviations: GO: gene ontology; BP: biological process; CC: cell. Component; MF: molecular function. (DOCX) [file pone.0060447.s006.docx]

Table S6. Functional annotation chart: Most relevant biological terms associated with HFS.

| Category | Term | Number of genes | P-Value | Benjamini |
| --- | --- | --- | --- | --- |
| GOTERM_BP | response to endogenous stimulus | 12 | 3,00E-05 | 3,30E-02 |
| GOTERM_BP | response to hormone stimulus | 11 | 6,20E-05 | 3,30E-02 |
| GOTERM_BP | ion transport | 12 | 1,20E-04 | 4,30E-02 |
| GOTERM_BP | response to organic substance | 14 | 1,40E-04 | 3,70E-02 |
| GOTERM_BP | negative regulation of signal transduction | 7 | 2,30E-04 | 4,90E-02 |
| GOTERM_BP | regulation of odontogenesis of dentine-containing tooth | 3 | 2,80E-04 | 4,90E-02 |
| GOTERM_BP | regulation of growth | 8 | 3,00E-04 | 4,60E-02 |
| GOTERM_BP | regulation of odontogenesis | 3 | 3,90E-04 | 5,20E-02 |
| GOTERM_BP | negative regulation of cell communication | 7 | 4,80E-04 | 5,70E-02 |
| GOTERM_BP | positive regulation of apoptosis | 8 | 5,50E-04 | 5,90E-02 |
| GOTERM_BP | positive regulation of programmed cell death | 8 | 5,70E-04 | 5,50E-02 |
| GOTERM_BP | positive regulation of cell death | 8 | 6,10E-04 | 5,40E-02 |
| GOTERM_BP | regulation of synaptic transmission | 6 | 7,50E-04 | 6,10E-02 |
| GOTERM_BP | regulation of systemic arterial blood pressure | 4 | 8,70E-04 | 6,50E-02 |
| GOTERM_BP | regulation of transmission of nerve impulse | 6 | 1,00E-03 | 7,10E-02 |
| GOTERM_BP | negative regulation of growth | 5 | 1,00E-03 | 6,70E-02 |
| GOTERM_BP | response to temperature stimulus | 5 | 1,10E-03 | 6,50E-02 |
| GOTERM_BP | regulation of blood pressure | 5 | 1,20E-03 | 6,90E-02 |
| GOTERM_BP | positive regulation of heart rate | 3 | 1,20E-03 | 6,70E-02 |
| GOTERM_BP | cation transport | 9 | 1,30E-03 | 6,60E-02 |
| GOTERM_BP | regulation of neurological system process | 6 | 1,40E-03 | 6,80E-02 |
| GOTERM_BP | calcium ion transport | 5 | 1,70E-03 | 8,30E-02 |
| GOTERM_BP | death | 8 | 2,20E-03 | 1,00E-01 |
| GOTERM_BP | positive regulation of heart contraction | 3 | 2,50E-03 | 1,10E-01 |
| GOTERM_BP | regulation of apoptosis | 10 | 2,50E-03 | 1,00E-01 |
| GOTERM_BP | regulation of programmed cell death | 10 | 2,70E-03 | 1,10E-01 |
| GOTERM_BP | regulation of heart contraction | 4 | 2,70E-03 | 1,00E-01 |
| GOTERM_BP | regulation of cell death | 10 | 2,80E-03 | 1,00E-01 |
| GOTERM_BP | positive regulation of catalytic activity | 8 | 3,10E-03 | 1,10E-01 |
| GOTERM_BP | regulation of insulin receptor signaling pathway | 3 | 3,80E-03 | 1,30E-01 |
| GOTERM_BP | di-, tri-valent inorganic cation transport | 5 | 3,90E-03 | 1,30E-01 |
| GOTERM_BP | circulatory system process | 5 | 4,70E-03 | 1,50E-01 |
| GOTERM_BP | blood circulation | 5 | 4,70E-03 | 1,50E-01 |
| GOTERM_BP | regulation of BMP signaling pathway | 3 | 4,90E-03 | 1,50E-01 |
| GOTERM_BP | regulation of heart rate | 3 | 5,30E-03 | 1,60E-01 |
| GOTERM_BP | regulation of catecholamine secretion | 3 | 5,30E-03 | 1,60E-01 |
| GOTERM_CC | extracellular region part | 10 | 5,40E-03 | 5,70E-01 |
| GOTERM_BP | positive regulation of molecular function | 8 | 6,30E-03 | 1,80E-01 |
| GOTERM_BP | response to ethanol | 4 | 7,10E-03 | 1,90E-01 |
| GOTERM_CC | cell projection part | 6 | 7,40E-03 | 4,30E-01 |
| GOTERM_BP | regulation of systemic arterial blood pressure mediated by a chemical signal | 3 | 7,60E-03 | 2,00E-01 |
| GOTERM_BP | cellular metal ion homeostasis | 5 | 7,60E-03 | 2,00E-01 |
| GOTERM_BP | response to abiotic stimulus | 7 | 7,70E-03 | 1,90E-01 |
| GOTERM_BP | metal ion transport | 7 | 8,10E-03 | 2,00E-01 |
| GOTERM_BP | metal ion homeostasis | 5 | 8,70E-03 | 2,10E-01 |
| GOTERM_BP | cell death | 7 | 9,10E-03 | 2,10E-01 |
| GOTERM_BP | positive regulation of blood pressure | 3 | 9,10E-03 | 2,10E-01 |
| GOTERM_BP | positive regulation of cellular component organization | 5 | 9,20E-03 | 2,00E-01 |
| GOTERM_BP | induction of apoptosis | 5 | 9,80E-03 | 2,10E-01 |
| GOTERM_BP | induction of programmed cell death | 5 | 9,80E-03 | 2,10E-01 |
| GOTERM_BP | regulation of amine transport | 3 | 1,00E-02 | 2,20E-01 |
| GOTERM_BP | regulation of system process | 6 | 1,30E-02 | 2,60E-01 |
| GOTERM_BP | positive regulation of muscle hypertrophy | 2 | 1,30E-02 | 2,60E-01 |
| GOTERM_BP | positive regulation of odontogenesis of dentine-containing tooth | 2 | 1,30E-02 | 2,60E-01 |
| GOTERM_BP | positive regulation of odontogenesis | 2 | 1,30E-02 | 2,60E-01 |
| GOTERM_BP | positive regulation of cardiac muscle hypertrophy | 2 | 1,30E-02 | 2,60E-01 |
| GOTERM_BP | response to organic cyclic substance | 5 | 1,40E-02 | 2,60E-01 |
| GOTERM_BP | cellular homeostasis | 7 | 1,40E-02 | 2,60E-01 |
| GOTERM_BP | induction of apoptosis by extracellular signals | 3 | 1,50E-02 | 2,70E-01 |
| GOTERM_BP | regulation of MAPKKK cascade | 4 | 1,50E-02 | 2,70E-01 |
| GOTERM_BP | response to peptide hormone stimulus | 5 | 1,60E-02 | 2,80E-01 |
| GOTERM_CC | extracellular region | 13 | 1,70E-02 | 5,80E-01 |
| GOTERM_BP | tissue morphogenesis | 5 | 1,70E-02 | 3,00E-01 |
| GOTERM_BP | activation of phospholipase C activity | 3 | 1,90E-02 | 3,10E-01 |
| GOTERM_BP | positive regulation of phospholipase C activity | 3 | 1,90E-02 | 3,10E-01 |
| GOTERM_BP | cellular cation homeostasis | 5 | 1,90E-02 | 3,10E-01 |
| GOTERM_BP | regulation of blood vessel size | 3 | 1,90E-02 | 3,10E-01 |
| GOTERM_BP | regulation of tube size | 3 | 1,90E-02 | 3,10E-01 |
| GOTERM_BP | anion transport | 4 | 2,00E-02 | 3,10E-01 |
| GOTERM_MF | hormone activity | 4 | 2,00E-02 | 9,90E-01 |
| GOTERM_BP | metanephros development | 3 | 2,00E-02 | 3,10E-01 |
| GOTERM_MF | channel regulator activity | 3 | 2,00E-02 | 8,80E-01 |
| GOTERM_BP | positive regulation of phospholipase activity | 3 | 2,10E-02 | 3,20E-01 |
| GOTERM_BP | vascular process in circulatory system | 3 | 2,10E-02 | 3,20E-01 |
| GOTERM_BP | regulation of protein kinase cascade | 5 | 2,20E-02 | 3,30E-01 |
| GOTERM_BP | apoptosis | 6 | 2,20E-02 | 3,20E-01 |
| GOTERM_BP | regulation of secretion | 5 | 2,20E-02 | 3,20E-01 |
| GOTERM_BP | positive regulation of hydrolase activity | 4 | 2,20E-02 | 3,20E-01 |
| GOTERM_BP | cellular ion homeostasis | 6 | 2,30E-02 | 3,20E-01 |
| GOTERM_BP | regulation of phospholipase activity | 3 | 2,30E-02 | 3,20E-01 |
| GOTERM_BP | positive regulation of multicellular organismal process | 5 | 2,30E-02 | 3,20E-01 |
| GOTERM_BP | cellular chemical homeostasis | 6 | 2,40E-02 | 3,20E-01 |
| GOTERM_BP | cellular response to hormone stimulus | 4 | 2,40E-02 | 3,20E-01 |
| GOTERM_BP | programmed cell death | 6 | 2,40E-02 | 3,10E-01 |
| GOTERM_BP | positive regulation of lipase activity | 3 | 2,60E-02 | 3,30E-01 |
| GOTERM_BP | regulation of hair cycle | 2 | 2,60E-02 | 3,30E-01 |
| GOTERM_BP | regulation of hair follicle development | 2 | 2,60E-02 | 3,30E-01 |
| GOTERM_MF | type 1 angiotensin receptor binding | 2 | 2,70E-02 | 8,50E-01 |
| GOTERM_MF | angiotensin receptor binding | 2 | 2,70E-02 | 8,50E-01 |
| GOTERM_BP | cation homeostasis | 5 | 2,80E-02 | 3,40E-01 |
| GOTERM_CC | plasma membrane | 19 | 2,80E-02 | 6,60E-01 |
| GOTERM_BP | regulation of hydrolase activity | 5 | 2,80E-02 | 3,40E-01 |
| GOTERM_CC | extracellular space | 7 | 3,00E-02 | 6,10E-01 |
| GOTERM_BP | natriuresis | 2 | 3,00E-02 | 3,60E-01 |
| GOTERM_BP | regulation of cardiac muscle hypertrophy | 2 | 3,00E-02 | 3,60E-01 |
| GOTERM_BP | tube morphogenesis | 4 | 3,00E-02 | 3,60E-01 |
| GOTERM_BP | ion homeostasis | 6 | 3,10E-02 | 3,60E-01 |
| GOTERM_BP | negative regulation of multicellular organismal process | 4 | 3,20E-02 | 3,60E-01 |
| GOTERM_BP | regulation of cellular localization | 5 | 3,30E-02 | 3,70E-01 |
| GOTERM_BP | regulation of protein modification process | 5 | 3,40E-02 | 3,70E-01 |
| GOTERM_BP | regulation of muscle hypertrophy | 2 | 3,50E-02 | 3,80E-01 |
| GOTERM_BP | response to nutrient levels | 5 | 3,50E-02 | 3,70E-01 |
| GOTERM_BP | positive regulation of MAPKKK cascade | 3 | 3,50E-02 | 3,70E-01 |
| GOTERM_BP | cellular calcium ion homeostasis | 4 | 3,50E-02 | 3,70E-01 |
| GOTERM_BP | homeostatic process | 8 | 3,50E-02 | 3,70E-01 |
| GOTERM_BP | positive regulation of signal transduction | 5 | 3,60E-02 | 3,70E-01 |
| GOTERM_BP | calcium ion homeostasis | 4 | 3,70E-02 | 3,80E-01 |
| GOTERM_BP | second-messenger-mediated signaling | 4 | 3,80E-02 | 3,80E-01 |
| GOTERM_BP | regulation of cell growth | 4 | 3,80E-02 | 3,80E-01 |
| GOTERM_BP | response to steroid hormone stimulus | 5 | 3,80E-02 | 3,80E-01 |
| GOTERM_BP | positive regulation of protein kinase cascade | 4 | 3,80E-02 | 3,80E-01 |
| GOTERM_BP | regulation of lipase activity | 3 | 3,90E-02 | 3,80E-01 |
| GOTERM_BP | regulation of norepinephrine secretion | 2 | 3,90E-02 | 3,70E-01 |
| GOTERM_BP | regulation of protein amino acid phosphorylation | 4 | 3,90E-02 | 3,70E-01 |
| GOTERM_MF | calcium channel activity | 3 | 3,90E-02 | 8,70E-01 |
| GOTERM_BP | response to extracellular stimulus | 5 | 4,30E-02 | 4,00E-01 |
| GOTERM_BP | positive regulation of organelle organization | 3 | 4,40E-02 | 4,00E-01 |
| GOTERM_BP | cellular response to insulin stimulus | 3 | 4,50E-02 | 4,10E-01 |
| GOTERM_MF | adrenoceptor activity | 2 | 4,50E-02 | 8,50E-01 |
| GOTERM_MF | metal ion transmembrane transporter activity | 5 | 4,60E-02 | 8,10E-01 |
| GOTERM_BP | intracellular signaling cascade | 9 | 4,70E-02 | 4,20E-01 |
| GOTERM_BP | positive regulation of systemic arterial blood pressure | 2 | 4,70E-02 | 4,20E-01 |
| GOTERM_BP | membrane protein intracellular domain proteolysis | 2 | 4,70E-02 | 4,20E-01 |
| GOTERM_BP | regulation of cell size | 4 | 4,70E-02 | 4,10E-01 |
| GOTERM_BP | inorganic anion transport | 3 | 4,90E-02 | 4,20E-01 |
| GOTERM_BP | regulation of synaptic plasticity | 3 | 4,90E-02 | 4,20E-01 |
| GOTERM_BP | positive regulation of MAP kinase activity | 3 | 5,00E-02 | 4,30E-01 |

*Abbrevations: GO: gene ontology; BP: biological process; CC: cell. Component; MF: molecular function.*
