# Supplementary material for: Striatal Molecular Signature of Subchronic Subthalamic Nucleus High Frequency Stimulation in Parkinsonian Rat
Source: PLoS One. 2013 Apr 4;8(4):e60447. doi: 10.1371/journal.pone.0060447 (PMC3617149; doi:10.1371/journal.pone.0060447)
Supplement: Table S7 — Functional annotation chart: Most relevant biological terms associated with DOPA. (DOCX) [file pone.0060447.s007.docx]

Table S7. Functional annotation chart: Most relevant biological terms associated with DOPA.

| Category | Term | Number of genes | P-Value | Benjamini |
| --- | --- | --- | --- | --- |
| GOTERM_BP | feeding behavior | 7 | 2,60E-04 | 3,20E-01 |
| GOTERM_BP | B cell mediated immunity | 6 | 2,70E-04 | 1,80E-01 |
| GOTERM_BP | regulation of system process | 13 | 3,30E-04 | 1,50E-01 |
| GOTERM_BP | positive regulation of odontogenesis | 3 | 4,00E-04 | 1,40E-01 |
| GOTERM_BP | positive regulation of odontogenesis of dentine-containing tooth | 3 | 4,00E-04 | 1,40E-01 |
| GOTERM_BP | response to ethanol | 7 | 6,10E-04 | 1,70E-01 |
| GOTERM_BP | lymphocyte mediated immunity | 6 | 6,80E-04 | 1,60E-01 |
| GOTERM_BP | adaptive immune response | 6 | 9,80E-04 | 1,90E-01 |
| GOTERM_BP | adaptive immune response based on somatic recombination of immune receptors built from immunoglobulin superfamily domains | 6 | 9,80E-04 | 1,90E-01 |
| GOTERM_BP | leukocyte mediated immunity | 6 | 1,70E-03 | 2,80E-01 |
| GOTERM_BP | regulation of odontogenesis of dentine-containing tooth | 3 | 1,90E-03 | 2,70E-01 |
| GOTERM_BP | epithelium development | 10 | 2,10E-03 | 2,70E-01 |
| GOTERM_BP | urogenital system development | 8 | 2,20E-03 | 2,50E-01 |
| GOTERM_BP | immunoglobulin mediated immune response | 5 | 2,30E-03 | 2,40E-01 |
| GOTERM_BP | regulation of growth | 11 | 2,40E-03 | 2,40E-01 |
| GOTERM_BP | kidney development | 7 | 2,50E-03 | 2,30E-01 |
| GOTERM_BP | regulation of odontogenesis | 3 | 2,70E-03 | 2,30E-01 |
| GOTERM_BP | response to organic cyclic substance | 9 | 3,10E-03 | 2,50E-01 |
| GOTERM_BP | growth | 9 | 3,50E-03 | 2,70E-01 |
| GOTERM_BP | tissue morphogenesis | 9 | 4,90E-03 | 3,30E-01 |
| GOTERM_BP | eating behavior | 4 | 4,90E-03 | 3,20E-01 |
| GOTERM_BP | positive regulation of catalytic activity | 13 | 5,40E-03 | 3,30E-01 |
| GOTERM_BP | cellular hormone metabolic process | 5 | 6,00E-03 | 3,50E-01 |
| GOTERM_BP | response to hormone stimulus | 14 | 6,20E-03 | 3,40E-01 |
| GOTERM_BP | response to endogenous stimulus | 15 | 6,60E-03 | 3,50E-01 |
| GOTERM_BP | regulation of blood pressure | 6 | 7,70E-03 | 3,80E-01 |
| GOTERM_BP | regulation of ATPase activity | 3 | 8,10E-03 | 3,90E-01 |
| GOTERM_BP | blood circulation | 7 | 9,30E-03 | 4,10E-01 |
| GOTERM_BP | circulatory system process | 7 | 9,30E-03 | 4,10E-01 |
| GOTERM_BP | ossification | 6 | 9,70E-03 | 4,10E-01 |
| GOTERM_BP | behavior | 12 | 1,00E-02 | 4,20E-01 |
| GOTERM_BP | striated muscle contraction | 4 | 1,00E-02 | 4,10E-01 |
| GOTERM_BP | response to organic substance | 20 | 1,10E-02 | 4,10E-01 |
| GOTERM_BP | gliogenesis | 5 | 1,10E-02 | 4,00E-01 |
| GOTERM_BP | ureteric bud development | 4 | 1,10E-02 | 4,00E-01 |
| GOTERM_BP | regulation of apoptosis | 16 | 1,20E-02 | 4,10E-01 |
| GOTERM_BP | regulation of programmed cell death | 16 | 1,30E-02 | 4,40E-01 |
| GOTERM_BP | immune effector process | 6 | 1,30E-02 | 4,30E-01 |
| GOTERM_BP | regulation of cell death | 16 | 1,40E-02 | 4,30E-01 |
| GOTERM_BP | positive regulation of hormone secretion | 4 | 1,40E-02 | 4,40E-01 |
| GOTERM_BP | bone development | 6 | 1,50E-02 | 4,40E-01 |
| GOTERM_BP | positive regulation of molecular function | 13 | 1,50E-02 | 4,30E-01 |
| GOTERM_BP | positive regulation of MAP kinase activity | 5 | 1,50E-02 | 4,30E-01 |
| GOTERM_BP | blood vessel development | 8 | 1,80E-02 | 4,70E-01 |
| GOTERM_BP | negative regulation of cell communication | 8 | 1,80E-02 | 4,70E-01 |
| GOTERM_BP | response to abiotic stimulus | 11 | 1,80E-02 | 4,70E-01 |
| GOTERM_BP | response to corticosteroid stimulus | 6 | 1,80E-02 | 4,70E-01 |
| GOTERM_BP | response to steroid hormone stimulus | 9 | 2,00E-02 | 4,90E-01 |
| GOTERM_BP | metanephros development | 4 | 2,00E-02 | 4,90E-01 |
| GOTERM_BP | vasculature development | 8 | 2,00E-02 | 4,80E-01 |
| GOTERM_BP | blood vessel morphogenesis | 7 | 2,10E-02 | 4,90E-01 |
| GOTERM_BP | response to corticosterone stimulus | 3 | 2,20E-02 | 5,00E-01 |
| GOTERM_BP | positive regulation of protein kinase activity | 7 | 2,40E-02 | 5,20E-01 |
| GOTERM_BP | tube development | 8 | 2,50E-02 | 5,30E-01 |
| GOTERM_BP | MAPKKK cascade | 6 | 2,50E-02 | 5,30E-01 |
| GOTERM_BP | positive regulation of kinase activity | 7 | 2,80E-02 | 5,60E-01 |
| GOTERM_BP | negative regulation of locomotion | 4 | 2,80E-02 | 5,50E-01 |
| GOTERM_BP | negative regulation of signal transduction | 7 | 2,90E-02 | 5,50E-01 |
| GOTERM_BP | negative regulation of growth | 5 | 3,10E-02 | 5,70E-01 |
| GOTERM_BP | glial cell differentiation | 4 | 3,10E-02 | 5,60E-01 |
| GOTERM_BP | response to mineralocorticoid stimulus | 3 | 3,10E-02 | 5,60E-01 |
| GOTERM_BP | response to temperature stimulus | 5 | 3,20E-02 | 5,60E-01 |
| GOTERM_BP | activation of MAPK activity | 4 | 3,20E-02 | 5,60E-01 |
| GOTERM_BP | regulation of hydrolase activity | 8 | 3,30E-02 | 5,70E-01 |
| GOTERM_BP | cell-matrix adhesion | 4 | 3,40E-02 | 5,70E-01 |
| GOTERM_BP | embryonic morphogenesis | 9 | 3,40E-02 | 5,60E-01 |
| GOTERM_BP | skeletal system development | 8 | 3,40E-02 | 5,60E-01 |
| GOTERM_BP | positive regulation of transferase activity | 7 | 3,40E-02 | 5,50E-01 |
| GOTERM_BP | negative regulation of multicellular organismal process | 6 | 3,70E-02 | 5,70E-01 |
| GOTERM_BP | epithelial cell differentiation | 5 | 3,80E-02 | 5,80E-01 |
| GOTERM_BP | hormone metabolic process | 5 | 3,80E-02 | 5,80E-01 |
| GOTERM_BP | response to cAMP | 4 | 3,80E-02 | 5,80E-01 |
| GOTERM_BP | regulation of protein kinase activity | 8 | 3,80E-02 | 5,70E-01 |
| GOTERM_BP | intracellular signaling cascade | 18 | 3,90E-02 | 5,70E-01 |
| GOTERM_BP | vitamin A metabolic process | 3 | 3,90E-02 | 5,70E-01 |
| GOTERM_BP | embryonic organ development | 7 | 4,00E-02 | 5,70E-01 |
| GOTERM_BP | negative regulation of apoptosis | 9 | 4,00E-02 | 5,70E-01 |
| GOTERM_BP | regulation of muscle contraction | 4 | 4,10E-02 | 5,70E-01 |
| GOTERM_BP | negative regulation of programmed cell death | 9 | 4,30E-02 | 5,80E-01 |
| GOTERM_BP | regulation of synaptic transmission | 6 | 4,30E-02 | 5,80E-01 |
| GOTERM_BP | negative regulation of cell death | 9 | 4,30E-02 | 5,80E-01 |
| GOTERM_BP | regulation of MAP kinase activity | 5 | 4,40E-02 | 5,80E-01 |
| GOTERM_BP | negative regulation of cardiac muscle cell proliferation | 2 | 4,60E-02 | 5,90E-01 |
| GOTERM_BP | positive regulation of gamma-aminobutyric acid secretion | 2 | 4,60E-02 | 5,90E-01 |
| GOTERM_BP | positive regulation of amino acid transport | 2 | 4,60E-02 | 5,90E-01 |
| GOTERM_BP | eye morphogenesis | 4 | 4,60E-02 | 5,90E-01 |
| GOTERM_BP | retinoid metabolic process | 3 | 4,70E-02 | 5,90E-01 |
| GOTERM_BP | diterpenoid metabolic process | 3 | 4,70E-02 | 5,90E-01 |
| GOTERM_BP | regulation of kinase activity | 8 | 4,80E-02 | 5,90E-01 |
| GOTERM_BP | neuropeptide signaling pathway | 4 | 4,90E-02 | 6,00E-01 |
| GOTERM_BP | positive regulation of secretion | 5 | 4,90E-02 | 6,00E-01 |
| GOTERM_BP | catechol metabolic process | 3 | 5,00E-02 | 5,90E-01 |
| GOTERM_BP | response to cold | 3 | 5,00E-02 | 5,90E-01 |
| GOTERM_BP | catecholamine metabolic process | 3 | 5,00E-02 | 5,90E-01 |
| GOTERM_BP | diol metabolic process | 3 | 5,00E-02 | 5,90E-01 |
| GOTERM_BP | response to light stimulus | 5 | 5,10E-02 | 6,00E-01 |
| GOTERM_BP | learning | 4 | 5,10E-02 | 5,90E-01 |
| GOTERM_BP | immune response | 10 | 5,10E-02 | 5,90E-01 |
| GOTERM_BP | wound healing | 6 | 5,20E-02 | 5,90E-01 |
| GOTERM_BP | cell-substrate adhesion | 4 | 5,30E-02 | 5,90E-01 |
| GOTERM_BP | phenol metabolic process | 3 | 5,30E-02 | 5,90E-01 |
| GOTERM_BP | transcription | 14 | 5,40E-02 | 5,90E-01 |
| GOTERM_BP | regulation of transmission of nerve impulse | 6 | 5,40E-02 | 5,90E-01 |
| GOTERM_BP | positive regulation of blood pressure | 3 | 5,60E-02 | 6,00E-01 |
| GOTERM_BP | terpenoid metabolic process | 3 | 5,60E-02 | 6,00E-01 |
| GOTERM_BP | response to glucocorticoid stimulus | 5 | 5,60E-02 | 6,00E-01 |
| GOTERM_BP | muscle filament sliding | 2 | 5,70E-02 | 6,00E-01 |
| GOTERM_BP | regulation of gamma-aminobutyric acid secretion | 2 | 5,70E-02 | 6,00E-01 |
| GOTERM_BP | actin-myosin filament sliding | 2 | 5,70E-02 | 6,00E-01 |
| GOTERM_BP | regulation of transferase activity | 8 | 5,90E-02 | 6,10E-01 |
| GOTERM_BP | response to mechanical stimulus | 4 | 6,00E-02 | 6,10E-01 |
| GOTERM_BP | developmental growth | 5 | 6,10E-02 | 6,20E-01 |
| GOTERM_BP | regulation of amine transport | 3 | 6,20E-02 | 6,20E-01 |
| GOTERM_BP | fat-soluble vitamin metabolic process | 3 | 6,50E-02 | 6,30E-01 |
| GOTERM_BP | regulation of vasoconstriction | 3 | 6,50E-02 | 6,30E-01 |
| GOTERM_BP | regulation of secretion | 7 | 6,50E-02 | 6,30E-01 |
| GOTERM_BP | G-protein signaling, coupled to cyclic nucleotide second messenger | 4 | 6,60E-02 | 6,30E-01 |
| GOTERM_BP | anion transport | 5 | 6,60E-02 | 6,20E-01 |
| GOTERM_BP | regulation of locomotion | 6 | 6,70E-02 | 6,30E-01 |
| GOTERM_BP | regulation of neurological system process | 6 | 6,70E-02 | 6,30E-01 |
| GOTERM_BP | actin-mediated cell contraction | 2 | 6,80E-02 | 6,30E-01 |
| GOTERM_BP | transmembrane receptor protein serine/threonine kinase signaling pathway | 4 | 7,20E-02 | 6,50E-01 |
| GOTERM_BP | induction of apoptosis | 6 | 7,20E-02 | 6,50E-01 |
| GOTERM_BP | induction of programmed cell death | 6 | 7,20E-02 | 6,50E-01 |
| GOTERM_BP | multicellular organismal homeostasis | 4 | 7,40E-02 | 6,50E-01 |
| GOTERM_BP | regulation of hormone secretion | 4 | 7,60E-02 | 6,60E-01 |
| GOTERM_BP | positive regulation of hydrolase activity | 5 | 7,60E-02 | 6,60E-01 |
| GOTERM_BP | muscle contraction | 4 | 7,80E-02 | 6,60E-01 |
| GOTERM_BP | regulation of smooth muscle contraction | 3 | 7,80E-02 | 6,60E-01 |
| GOTERM_BP | cellular response to hormone stimulus | 5 | 8,10E-02 | 6,70E-01 |
| GOTERM_BP | activation of phospholipase C activity by G-protein coupled receptor protein signaling pathway coupled to IP3 second messenger | 3 | 8,10E-02 | 6,70E-01 |
| GOTERM_BP | neuron differentiation | 10 | 8,40E-02 | 6,80E-01 |
| GOTERM_BP | anti-apoptosis | 5 | 8,80E-02 | 6,90E-01 |
| GOTERM_BP | regulation of systemic arterial blood pressure | 3 | 8,80E-02 | 6,90E-01 |
| GOTERM_BP | positive regulation of apoptosis | 8 | 8,90E-02 | 6,90E-01 |
| GOTERM_BP | biological adhesion | 10 | 8,90E-02 | 6,90E-01 |
| GOTERM_BP | cell adhesion | 10 | 8,90E-02 | 6,90E-01 |
| GOTERM_BP | regulation of amino acid transport | 2 | 8,90E-02 | 6,90E-01 |
| GOTERM_BP | estrogen metabolic process | 2 | 8,90E-02 | 6,90E-01 |
| GOTERM_BP | embryonic organ morphogenesis | 5 | 8,90E-02 | 6,80E-01 |
| GOTERM_BP | response to reactive oxygen species | 4 | 9,10E-02 | 6,90E-01 |
| GOTERM_BP | positive regulation of programmed cell death | 8 | 9,10E-02 | 6,80E-01 |
| GOTERM_BP | response to inorganic substance | 7 | 9,10E-02 | 6,80E-01 |
| GOTERM_BP | camera-type eye morphogenesis | 3 | 9,20E-02 | 6,80E-01 |
| GOTERM_BP | organic anion transport | 3 | 9,20E-02 | 6,80E-01 |
| GOTERM_BP | regulation of phosphorylation | 9 | 9,20E-02 | 6,80E-01 |
| GOTERM_BP | cellular di-, tri-valent inorganic cation homeostasis | 6 | 9,30E-02 | 6,80E-01 |
| GOTERM_BP | positive regulation of cell death | 8 | 9,40E-02 | 6,80E-01 |
| GOTERM_BP | biogenic amine metabolic process | 4 | 9,50E-02 | 6,90E-01 |
| GOTERM_BP | regulation of muscle development | 3 | 9,50E-02 | 6,80E-01 |
| GOTERM_BP | cyclic-nucleotide-mediated signaling | 4 | 9,80E-02 | 6,90E-01 |
| GOTERM_BP | transforming growth factor beta receptor signaling pathway | 3 | 9,90E-02 | 6,90E-01 |
| GOTERM_BP | positive regulation of organic acid transport | 2 | 1,00E-01 | 6,90E-01 |
