# Supplementary material for: Striatal Molecular Signature of Subchronic Subthalamic Nucleus High Frequency Stimulation in Parkinsonian Rat
Source: PLoS One. 2013 Apr 4;8(4):e60447. doi: 10.1371/journal.pone.0060447 (PMC3617149; doi:10.1371/journal.pone.0060447)
Supplement: Table S8 — Functional annotation chart: Most relevant biological terms associated with DOPA/HFS. (DOCX) [file pone.0060447.s008.docx]

Table S8. Functional annotation chart: Most relevant biological terms associated with DOPA/HFS.

| Category | Term | Number of genes | P-Value | Benjamini |
| --- | --- | --- | --- | --- |
| GOTERM_BP | immunoglobulin mediated immune response | 3 | 2,50E-03 | 6,60E-01 |
| GOTERM_BP | B cell mediated immunity | 3 | 2,70E-03 | 4,40E-01 |
| GOTERM_BP | lymphocyte mediated immunity | 3 | 4,10E-03 | 4,40E-01 |
| GOTERM_BP | adaptive immune response based on somatic recombination of immune receptors built from immunoglobulin superfamily domains | 3 | 4,70E-03 | 3,90E-01 |
| GOTERM_BP | adaptive immune response | 3 | 4,70E-03 | 3,90E-01 |
| GOTERM_BP | leukocyte mediated immunity | 3 | 6,10E-03 | 4,00E-01 |
| GOTERM_BP | regulation of transcription | 8 | 8,50E-03 | 4,50E-01 |
| GOTERM_BP | immune effector process | 3 | 1,50E-02 | 6,00E-01 |
| GOTERM_BP | response to abiotic stimulus | 4 | 2,30E-02 | 7,10E-01 |
| GOTERM_BP | regulation of transcription, DNA-dependent | 6 | 3,70E-02 | 8,30E-01 |
| GOTERM_BP | regulation of RNA metabolic process | 6 | 4,00E-02 | 8,20E-01 |
| GOTERM_BP | response to organic substance | 5 | 5,30E-02 | 8,80E-01 |
| GOTERM_BP | regulation of system process | 3 | 8,80E-02 | 9,60E-01 |
| GOTERM_BP | transcription | 4 | 8,90E-02 | 9,50E-01 |
